# Long-read sequencing of CYP2D6 may improve psychotropic prescribing and treatment outcomes: A systematic review and meta-analysis

**DOI:** 10.1177/02698811241268899

**Published:** 2024-09-11

**Authors:** Dean Kaptsis, Martin Lewis, Michael Sorich, Malcolm Battersby

**Affiliations:** 1 College of Medicine and Public Health, Flinders University, Bedford Park, SA, Australia; 2 Neuropsychiatric Laboratory, South Australian Health and Medical Research Institute, Adelaide, SA, Australia; 3 School of Biological Sciences, The University of Adelaide, Adelaide, SA, Australia

**Keywords:** CYP2D6, long-read sequencing, precision medicine, pharmacogenetics, psychotropic drugs

## Abstract

**Background:**

The enzyme expression (i.e. phenotype) of the Cytochrome P450 2D6 (CYP2D6) gene is highly relevant to the metabolism of psychotropic medications, and therefore to precision medicine (i.e. personalised prescribing).

**Aims:**

This review aims to assess the improvement in CYP2D6 phenotyping sensitivity (IPS) and accuracy (IPA) offered by long-read sequencing (LRS), a new genetic testing technology.

**Methods:**

Human DNA samples that underwent LRS genotyping of CYP2D6 in published, peer-reviewed clinical research were eligible for inclusion. A systematic literature search was conducted until 30 September 2023. CYP2D6 genotypes were translated into phenotypes using the international consensus method. IPS was the percentage of non-normal LRS CYP2D6 phenotypes undetectable with FDA-approved testing (AmpliChip). IPA was the percentage of LRS CYP2D6 phenotypes mischaracterised by non-LRS genetic tests (for samples with LRS and non-LRS data).

**Results:**

Six studies and 1411 samples were included. In a meta-analysis of four studies, IPS was 10% overall (95% CI = (2, 18); *n* = 1385), 20% amongst Oceanians (95% CI = (17, 23); *n* = 582) and 2% amongst Europeans (95% CI = (1, 4); *n* = 803). IPA was 4% in a large European cohort (95% CI = (2, 7); *n* = 567). When LRS was used selectively (e.g. for novel or complex CYP2D6 genotypes), very high figures were observed for IPS (e.g. 88%; 95% CI = (72, 100); *n* = 17; country = Japan) and IPA (e.g. 76%; 95% CI = (55, 98); *n* = 17; country = Japan).

**Conclusions:**

LRS improves CYP2D6 phenotyping compared to established genetic tests, particularly amongst Oceanian and Japanese individuals, and those with novel or complex genotypes. LRS may therefore assist in optimising personalised prescribing of psychotropic medications. Further research is needed to determine associated clinical benefits, such as increased medication safety and efficacy.

## Introduction

The Cytochrome P450 2D6 (CYP2D6) enzyme contributes to the metabolism of approximately 25% of prescribed medications and is particularly relevant to psychotropic medications (Zhou, 2009). Variability in CYP2D6 genotype is related to enzyme activity (i.e. metaboliser phenotype), predicting important pharmacological outcomes (Bertilsson et al., 2002). For example, CYP2D6 enzyme activity inversely relates to the plasma concentration of several typical antipsychotics (e.g. haloperidol), atypical antipsychotics (e.g. risperidone, aripiprazole), selective serotonin reuptake inhibitors (e.g. fluvoxamine) and tricyclic antidepressants (e.g. amitriptyline). Hence, subnormal enzyme activity increases the risk of adverse effects, and supernormal enzyme activity increases the risk of nonresponse (Beunk et al., 2023; Bousman et al., 2023; Hicks et al., 2017). In mental healthcare settings, two-thirds of patients may be prescribed a medication with a known variable response according to CYP2D6 metaboliser phenotype (Jameson et al., 2024). Standard dosing, without reference to CYP2D6 genotype, may therefore contribute to the limited success of antipsychotic clinical trials (51% response rate; Leucht et al., 2017) and first antidepressant trials (37% remission rate; Rush et al., 2006). Conversely, personalised prescribing based on CYP2D6 is recommended by several international guidelines (e.g. United States Food and Drug Administration (FDA); Clinical Pharmacogenetics Implementation Consortium (CPIC); Bousman et al., 2020) and has been shown to improve outcomes in the treatment of depression (Arnone et al., 2023).

Despite the apparent benefits of CYP2D6 genotyping, trial-and-error psychotropic prescribing remains predominant in many countries, including Australia and the United Kingdom (Jameson et al., 2024; NICE, 2022; Therapeutic Guidelines, 2021). The slow uptake of personalised prescribing may reflect concerns about the feasibility, accuracy and clinical utility of CYP2D6 testing (Bousman et al., 2020; Cavallari et al., 2019). The cost of pharmacogenetic testing is frequently cited as a barrier by both patients and clinicians, along with a lack of funding from third parties (e.g. health insurers; Jameson et al., 2021). Infrastructural barriers have also been identified, including a lack of available testing services and difficulty integrating test results into existing clinical information systems (Pearce et al., 2022; Qureshi et al., 2022). Meanwhile, existing trials of CYP2D6 genotype-guided prescribing have produced inconsistent findings, contributing to doubts about the readiness of pharmacogenetic testing for routine clinical practice (Barlati et al., 2023).

The benefits of CYP2D6 testing may be hindered by the complex and variable nature of the gene, rendering it difficult to genotype. CYP2D6 is repetitive, highly polymorphic, and characterised by a range of small and large variants which affect metaboliser profiles (Gaedigk, 2013). CYP2D6 variant profiles differ markedly by population, producing a wide range of normal (67%–90%), slower than normal (1%–16%) and faster than normal (1%–21%) metaboliser phenotypes across major ethnic groups (Gaedigk et al., 2017). To facilitate clinical testing, CYP2D6 genotypes are labelled using star (*) allele nomenclature, which indicates the variants and enzyme function associated with each allele (e.g. *1 predicts normal function and *4 predicts no function). The overall genotype (e.g. *1/*4, representing the alleles present on each chromosome) is used to predict metaboliser phenotype, with categories including ‘poor’, ‘intermediate’, ‘normal’, ‘ultrarapid’ and ‘indeterminate’ (Caudle et al., 2020). Metaboliser phenotype may then be used for personalised medication selection and dosing based on pharmacogenetics guidelines (Beunk et al., 2023; Bousman et al., 2023; Hicks et al., 2017). Sensitive and accurate genotyping of CYP2D6 is therefore crucial for effective phenotyping and precision medicine.

Established genetic tests may have limited CYP2D6 genotyping sensitivity and accuracy. Sanger sequencing, targeted genotyping, microarray and short-read sequencing (SRS) have typically been used to genotype CYP2D6 in clinical populations (Fleeman et al., 2011; Pratt et al., 2021). Microarray and SRS offer high-throughput testing (Dodgen et al., 2013; Yang et al., 2017), making them affordable options for routine clinical use. However, the repetitive regions of CYP2D6 often exceed the read lengths of SRS, compromising test accuracy and the ability to distinguish CYP2D6 from its pseudogenes (Twist et al., 2016; Yang et al., 2017). Meanwhile, the Roche AmpliChip CYP450 microarray (henceforth AmpliChip), one of two FDA-approved CYP2D6 tests, can detect only 20 of the more than 100 currently recognised star alleles (Gaedigk et al., 2021; Lyon et al., 2012). The other FDA-approved test, xTAG CYP2D6 Kit v3, is capable of detecting only 16 star alleles, with none being unique from AmpliChip (Lyon et al., 2012). The star alleles not detected by AmpliChip and xTAG are more prevalent in non-European regions, such as Africa, Asia and Oceania (Gaedigk et al., 2017), limiting the usefulness of these tests across populations. Established tests may therefore mischaracterise or fail to detect CYP2D6 genotypes, leading to ineffective phenotyping and subsequent harms (e.g. over- or underdosing of medication).

Long-read sequencing (LRS), a newer genotyping technology, may overcome several challenges associated with CYP2D6. Though generally more expensive than SRS (Espinosa et al., 2024), LRS offers read lengths that span the entire CYP2D6 gene (Hu et al., 2021). Hence, LRS can resolve structural variations (e.g. large insertions and deletions) and lengthy, complex or repetitive genetic sequences (Goodwin et al., 2016). LRS has been posited as a gold standard genotyping technology (Kovaka et al., 2023) and has outperformed established technologies in preliminary testing of CYP2D6 (Buermans et al., 2017; Qiao et al., 2016; Yang et al., 2017). However, it is unclear whether LRS testing of CYP2D6 offers clinical benefit, as improved genotyping does not necessarily translate into improved phenotyping and precision medicine. There are several CYP2D6 genotypes associated with each metaboliser phenotype (e.g. *3/*4 and *5/*6 both predict a ‘poor’ metaboliser), so even if LRS detects different genotypes than established tests, there may be no indicated changes to prescribing. Medication or dosage change recommendations, according to prescribing guidelines (e.g. Bousman et al., 2023), can only arise if LRS predicts different phenotypes than established tests. The extent to which LRS improves CYP2D6 phenotyping therefore has serious implications for clinical CYP2D6 testing and precision medicine.

Existing reviews have addressed various aspects of CYP2D6 testing, including its evolution across time (Lauschke and Ingelman-Sundberg, 2019; Yang et al., 2017), impact in the clinical setting (Taylor et al., 2020), utility in guiding psychotropic pharmacotherapy (Arnone et al., 2023; Bousman et al., 2020; Islam et al., 2021), and prediction of psychotropic medication exposure and adverse reactions (Bousman et al., 2020; Milosavljevic et al., 2021). However, no reviews have investigated the extent to which LRS offers an improvement in CYP2D6 phenotyping compared to established genetic tests. To address this gap, the current review aimed to examine the percentage of LRS CYP2D6 metaboliser phenotypes that are not categorised as ‘normal’ and would be undetectable with the FDA-approved AmpliChip test (i.e. improvement in CYP2D6 phenotyping sensitivity). This review also aimed to examine the percentage of LRS CYP2D6 metaboliser phenotypes that differ from those derived from established genetic tests (i.e. improvement in CYP2D6 phenotyping accuracy).

## Methods

This systematic review and meta-analysis, which was pre-registered with PROSPERO (number: CRD42023433050), examined original, clinical research studies which used LRS to genotype CYP2D6 in human DNA samples. The purpose of this review was to examine the improvements in CYP2D6 phenotyping offered by LRS. This review was conducted in line with the Preferred Reporting Items for Systematic Reviews and Meta-Analyses (PRISMA) Statement (see Supplemental Tables S1 and S2).

### Eligibility criteria

Study and sample eligibility criteria are shown in Table 1. No restrictions were applied based on setting, language, publication date, specific LRS test used, specific non-LRS test used or utilisation of reference testing (e.g. Sanger sequencing). To minimise bias and maximise power, studies were excluded if they contained no unique clinical samples compared to another, larger included study. For all included studies, reference samples (i.e. samples drawn from a biorepository) were excluded to increase the relevance of findings to clinical CYP2D6 testing, and to minimise the potential for bias (e.g. testing of samples with known genotypes).

**Table 1.** Study and sample eligibility criteria.

Level	Domain	Inclusion criteria	Exclusion criteria
Study	Publication	Published and peer-reviewed	Unpublished or not peer reviewed
	Design	Quantitative	Qualitative
	Type	Original clinical research^a^ that used LRS to genotype CYP2D6	No clinical research^a^ (e.g. only tested repository DNA samples) or no LRS genotyping of CYP2D6
	Uniqueness	Contained one or more unique samples compared to other included studies	Contained no unique samples compared to another, larger included study
Sample	Profile	DNA samples from healthy or unhealthy human participant	Non-human DNA sample (e.g. animal or microbe DNA)
	Genotyping	Underwent CYP2D6 genotyping with LRS^b^	Did not undergo CYP2D6 genotyping with LRS
	Source	Original clinical research^a^	Biorepository
	Uniqueness	Not analysed in another, larger included study	Analysed in another, larger included study

LRS: long-read sequencing.

a Clinical research was defined as that which investigated diagnostic or treatment outcomes in participants drawn from a specified healthcare population (e.g. patients receiving tamoxifen chemotherapy).

b Samples were also eligible if they underwent CYP2D6 genotyping with non-LRS technology in addition to LRS technology (i.e. dual genotyping).

Where available, eligible outcomes data for each sample included CYP2D6 genotype (using star (*) allele nomenclature; Caudle et al., 2020), activity score and metaboliser phenotype pertaining to any LRS and non-LRS testing. All included samples were relevant to the examination of improvement in phenotyping sensitivity offered by LRS, as this only required checking LRS CYP2D6 genotypes for their detectability with AmpliChip (see Table 2). However, only samples with both LRS and non-LRS genotypes were relevant to the examination of improvement in phenotyping accuracy offered by LRS, as this required comparison of CYP2D6 genotypes produced by the respective technologies.

**Table 2.** CYP2D6 star (*) alleles and duplication events detectable with the Roche AmpliChip CYP450 test (i.e. AmpliChip).

Test parameter	No.	Alleles
Detectable star alleles	20	*1, *2, *3, *4, *5, *6, *7, *8, *9, *10, *11, *15, *17, *19, *20, *29, *35, *36, *40, *41
Detectable duplication events	7	*1xN, *2*xN*, *4xN, *10xN, *17xN, *35xN, *41xN

Source: Adapted from Lyon et al. (2012) and United States Food and Drug Administration (FDA, 2005).

*N*: number of duplicate alleles present.

### Information sources and search strategy

A systematic literature search was conducted in PubMed, MEDLINE, the Cochrane Library and Scopus. The publication date was from database inception onwards. Search terms included ‘Cytochrome P-450 CYP2D6’, ‘single-molecule real-time sequencing’, ‘long read sequencing’, ‘long-read sequencing’ and ‘nanopore sequencing’ and were entered as medical subject headings (MeSH terms) where available. The full search terms used for each database can be found in Supplemental Table S3. Manual searches were also conducted in the reference lists of eligible studies, and on the Pharmacogene Variation Consortium (PharmVar) CYP2D6 website (Gaedigk et al., 2021), which lists studies credited with discovering novel CYP2D6 star alleles. All information sources were most recently searched on September 30, 2023.

### Selection process

All studies identified in the literature search were imported into EndNote (version 20.6) and then exported to an EndNote library file. The library file was uploaded to Covidence (Veritas, 2023), a web-based systematic review management platform that was used for study selection. In Stage 1 of study selection, two authors (D.K. and M.L.) independently screened study titles and abstracts, and voted ‘Yes’, ‘No’ or ‘Maybe’ for each study based on apparent alignment with eligibility criteria (see Table 1). In Stage 2 of study selection, the same two authors independently conducted a full-text review of studies that had progressed through Stage 1 and voted ‘Include’ or ‘Exclude’ for each study based on apparent alignment with eligibility criteria. Following study selection, the same two authors independently conducted sample selection, which involved marking individual samples for exclusion if they appeared to meet exclusion criteria or not meet inclusion criteria.

Prior to commencing the selection process, authors agreed upon how each eligibility criterion would be assessed (e.g. the presence of a biorepository reference number would constitute grounds for excluding a sample based on its source; see Table 1). Six instances of disagreement occurred in Stage 1 of study selection and were resolved by a third author (M.B.). There were no other instances of disagreement during study or sample selection. All authors were blinded to each other’s decisions.

### Data extraction

For each study, samples that satisfied eligibility criteria were considered for data extraction. However, data extraction only occurred for samples that met one or both of the following conditions: (1) one or more CYP2D6 star alleles were AmpliChip-undetectable (i.e. were not compatible with the list of star alleles detectable with AmpliChip; see Table 2) or (2) genotyping occurred with both LRS and non-LRS testing, and the resultant CYP2D6 genotypes were discordant (i.e. contained at least one different star allele). For the remainder of the included samples, LRS was not expected to produce improvements in phenotyping, as their LRS genotype was AmpliChip-detectable and, if comparison data were available, concordant with the non-LRS genotype. However, these samples were still counted and included in all relevant tables, figures and analyses. One author extracted the data (D.K.) and another author (M.L.) checked the data for completeness. Data were recorded in a spreadsheet.

Where available, data extraction included individual sample number/identifier, ancestry, cohort (e.g. if the sample was part of a specified clinical trial), LRS data (specific test used; CYP2D6 genotype using star (*) allele nomenclature; CYP2D6 activity score; predicted metaboliser phenotype), any non-LRS comparison test data (specific test used; CYP2D6 genotype using star (*) allele nomenclature; CYP2D6 activity score; predicted metaboliser phenotype) and any gene duplication test data (specific test used; copy number of duplicated CYP2D6 star (*) alleles). Individual sample data were not available for a single included study (Hitchman et al., 2022), but summary data were sufficient for determining the number and nature of CYP2D6 genotypes that were AmpliChip-undetectable, allowing review analyses to proceed.

### Quality assessment

The Quality Assessment of Diagnostic Accuracy Studies-2 (QUADAS-2; Whiting et al., 2011) and Quality Assessment of Diagnostic Accuracy Studies-Comparative (QUADAS-C; Yang et al., 2021) were used to assess the included studies. Two authors (D.K. and M.L.) independently assigned a classification of ‘high risk’, ‘low risk’ or ‘uncertain risk’ across various domains for each study with respect to risk of bias and applicability concerns (i.e. study compatibility with review objective). Disagreements occurred whilst assessing two studies and were resolved by a third author (M.B.). All authors were blinded to each other’s decisions. A quality appraisal of review findings was also conducted by considering the quality assessment of included studies along with the consistency (i.e. degree of heterogeneity) and precision (i.e. confidence intervals and sample sizes) of measured outcomes.

### Data synthesis

Data were tabulated for each study, including in the form of sample-level data (Supplemental Table S4) and data relevant to the two components of the review objective: improvement in CYP2D6 phenotyping sensitivity (Supplemental Tables S5–S7) and improvement in CYP2D6 phenotyping accuracy (Supplemental Table S8). The risk difference was used to determine improvement in sensitivity and, where non-LRS genotypes were available, improvement in accuracy demonstrated by LRS for each study. However, the terms ‘improvement in CYP2D6 phenotyping sensitivity’ (IPS) and ‘improvement in CYP2D6 phenotyping accuracy’ (IPA) were used in place of risk difference. The confidence interval was set to 95% for determining statistical significance.

Meta-analysis was used to synthesise improvement in CYP2D6 phenotyping sensitivity (IPS) findings across studies using a Mantel–Haenszel test of the overall effect. A random-effects model was used to anticipate possible cross-study variability in true effect associated with the use of different genetic tests (e.g. different brands or models of LRS tests). Between-study and between-subgroup heterogeneity were analysed using tau squared. Planned meta-analysis for improvement in CYP2D6 phenotyping accuracy (IPA) was not possible due to no more than one study being available for each unique comparison between LRS and a non-LRS technology (e.g. LRS vs microarray; LRS vs SRS). Publication bias was assessed by visually inspecting funnel plots for each review outcome (IPS and IPA), but regression tests were not conducted due to the small number of included publications. The software package Review Manager (RevMan; version 5.4.1) was used to conduct the meta-analysis and produce forest plots. The software package GraphPad Prism (version 9.5.1) was used to create pie charts illustrating descriptive data.

#### Sample-level data

Sample-level data included all items gathered during data extraction. Information tables hosted by the Pharmacogenomics Knowledgebase (PharmGKB; Whirl-Carrillo et al., 2021) were used to assess CYP2D6 genotype to phenotype translations made by studies for agreement with the consensus translation method (Caudle et al., 2020). Where disagreements were observed, the consensus method took precedence for this review. Minimum copy number (i.e. 2) was assumed for star alleles reported to have duplications but which did not specify the copy number (e.g. *1xN). Individual samples were annotated for the detectability of their LRS genotypes with AmpliChip (per Table 2), and for any differences in star alleles, activity score and metaboliser phenotype between LRS and non-LRS genotypes (where comparison data were available). CYP2D6 suballele-level data were not considered when making these determinations (e.g. *2.001 was not considered different from *2.002), as suballeles of a given star allele share the same functional status (Gaedigk et al., 2021).

#### Improvement in phenotyping sensitivity

The number and percentage of LRS CYP2D6 genotypes that were AmpliChip-undetectable (per Table 2) were counted and listed for each study. The genotypes were further categorised according to the predicted metaboliser phenotype, using the consensus genotype to phenotype translation method (Caudle et al., 2020). To determine the improvement in CYP2D6 phenotyping sensitivity (IPS) demonstrated by LRS, the following data were used: (1) the percentage of LRS CYP2D6 genotypes that were AmpliChip-detectable (AD) and (2) the percentage of LRS CYP2D6 genotypes that were AmpliChip-undetectable and predicted a non-normal metaboliser phenotype (NN). These data were subject to the following formula to produce a risk difference measure for each study, which was combined for meta-analysis: ((AD + NN) − AD) = IPS. Given that CYP2D6 allele frequencies vary significantly across global populations (Gaedigk et al., 2017), a subgroup analysis of studies by geographic region was included in the meta-analysis to examine differences in IPS across regions.

Non-normal CYP2D6 metaboliser phenotypes were defined as those that would be categorised as ‘poor’, ‘intermediate’, ‘ultrarapid’ or ‘indeterminate’ (Caudle et al., 2020). Though indeterminate phenotypes are based on CYP2D6 alleles with missing or only preliminary functional data, most alleles with preliminary data have thus far exhibited subnormal enzyme function (i.e. slow metabolism; Whirl-Carrillo et al., 2021). Several of these alleles are already considered by expert consensus to be of subnormal function (Caudle et al., 2020). Hence, indeterminate metaboliser phenotypes were considered non-normal for this review.

#### Improvement in phenotyping accuracy

The number and percentage of samples whose LRS and non-LRS CYP2D6 genotypes were discordant (i.e. contained one or more different star alleles) were counted and listed for each study that utilised both types of technologies. Discordant genotype pairs were further examined to see whether they predicted different CYP2D6 metaboliser phenotypes, with possible categories including ‘poor’, ‘intermediate’, ‘normal’, ‘ultrarapid’ and ‘indeterminate’ (Caudle et al., 2020). LRS-derived metaboliser phenotypes were also considered different if the respective non-LRS genotype was incomplete (i.e. unable to predict the non-LRS metaboliser phenotype). However, LRS-derived metaboliser phenotypes were not considered different if the respective LRS genotype was incomplete (i.e. unable to predict the LRS metaboliser phenotype). To determine the improvement in CYP2D6 phenotyping accuracy (IPA) demonstrated by LRS, the following data were used: (1) the percentage of samples with LRS and non-LRS CYP2D6 genotypes that were concordant (C) and (2) the percentage of samples with LRS and non-LRS CYP2D6 genotypes that were discordant and predicted different CYP2D6 metaboliser phenotypes (D). These data were subject to the following formula to produce a risk difference measure for each study: ((C + D) − C) = IPA.

## Results

### Study selection

**Figure 1.**
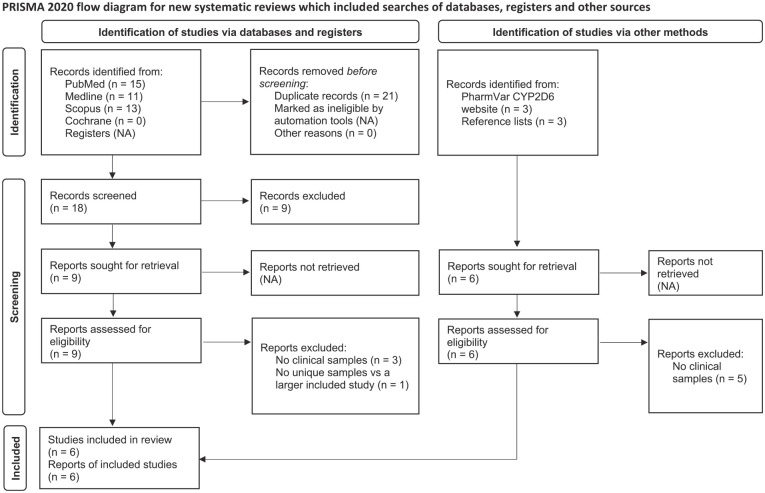
PRISMA flow diagram. PRISMA: Preferred Reporting Items for Systematic Reviews and Meta-Analyses.

The study selection process is detailed in Figure 1. Six independent studies were included, five of which were identified via database searches and one of which was identified via the Pharmacogene Variation Consortium (PharmVar) CYP2D6 website (Gaedigk et al., 2021).

### Study characteristics and outcomes data

Characteristics of the six included studies are presented in Table 3. There were 2560 samples in total (*N*), of which 1411 (55.1%) were included in the current review (*n*), on the basis that they were clinical samples genotyped with LRS. Most included samples were reported to be of European ancestry (56.9%) or Oceanian ancestry (39.3%; see Supplemental Table S9 for sample eligibility and ancestry details). Included samples were drawn from various clinical populations, including those defined by diagnosis or problem (Charnaud et al., 2022; Fukunaga et al., 2021; Liau et al., 2019), setting (Hitchman et al., 2022; Qiao et al., 2016), or a combination of diagnosis and treatment (van der Lee et al., 2021). Three studies were conducted in Oceania (*n* = 582), whilst the remaining three were conducted in Europe (*n* = 803), Asia (*n* = 17) and North America (*n* = 9). Four studies contained samples from specified clinical cohorts (e.g. clinical trials), and one of these studies listed which samples belonged to each independent cohort (van der Lee et al., 2021). Four studies used Pacific Biosciences LRS tests (*n* = 1184), and the remaining two used Oxford Nanopore Technology LRS tests (*n* = 227). Non-LRS tests were only used in four studies (*n* = 618), as were tests for gene duplication detection (*n* = 406). Types of non-LRS technology included SRS (Fukunaga et al., 2021), Sanger sequencing (Liau et al., 2019), microarray (van der Lee et al., 2021) and PCR with allele-specific primer extension (Qiao et al., 2016).

**Table 3.** Characteristics and outcomes data of included studies.

Study	Country	*N*	*n*	Clinical cohort(s)	Clinical sample description	Clinical sample ancestry^a^	LRS test	Non-LRS test	Duplication test	AmpliChip-undetectable LRS CYP2D6 genotypes^b^		Discordant LRS and non-LRS CYP2D6 genotypes^c^	
										Total (% of *n*)	Non-normal MPs^d^ (% of *n*)	Total (% of *n*)	Differing MPs^d^ (% of *n*)
Charnaud et al. (2022)	SB	387	355	Vivax cure trial	Malaria positive patients	Solomon Islander	PacBio Sequel I/II	Nil	TaqMan qPCR	81 (22.8)	74 (20.8)	NA	–
Fukunaga et al. (2021)	JP	990	17	Not specified	Psychiatric and cancer patients	Japanese	PacBio Sequel I	Illumina MiSeq	TaqMan qPCR	16 (94.1)	15 (88.2)	14 (82.4)	13 (76.5)
Hitchman et al. (2022)	NZ	202	202	GGDKANZ/PHS	Primary care patients	Pacific Islander (98.5%); European	ONT GridION	Nil	Nil	39 (19.3)	39 (19.3)	NA	–
Liau et al. (2019)	NZ	32	25	UDRUGS/GO-A	Adverse drug event patients	Not specified	ONT GridION	Sanger	Duplex PCR	4 (16.0)	4 (16.0)	1 (4.0)	1 (4.0)
Qiao et al. (2016)	US	38	9	Not specified	Healthy adult blood donors	Asian; European; Hispanic; African	PacBio RSII	xTAG v3	TaqMan qPCR	3 (33.3)	3 (33.3)	3 (33.3)	3 (33.3)
Van der Lee et al. (2021) ^e^	NL	608	567	CYPTAM	Tamoxifen patients (breast cancer)	European	PacBio RSII	AmpliChip	Nil	29 (5.1)	20 (3.5)	44 (77.6)	24 (4.2)
Van der Lee et al. (2021) ^e^	NL	225	167	CYPTAM-BRUT	Tamoxifen patients (breast cancer)	European	PacBio Sequel I	Nil	Nil	3 (1.8)	2 (1.2)	NA	–
Van der Lee et al. (2021) ^e^	NL	78	69	Venlafaxine	Venlafaxine patients	European	PacBio Sequel I	Nil	Nil	3 (4.3)	1 (1.4)	NA	–

*N*: total sample size; *n*: clinical samples genotyped with LRS; LRS: long-read sequencing; AmpliChip: Roche AmpliChip CYP450; MPs: metaboliser phenotypes; SB: Solomon Islands; PacBio: Pacific Biosciences; JP: Japan; NZ: New Zealand; GGDKANZ: Genetics of Gout, Diabetes, and Kidney Disease in Aotearoa New Zealand; PHS: Pasifika Heart Study; ONT: Oxford Nanopore Technology; UDRUGS: Understanding Adverse Drug Reactions Using Genomic Sequencing; GO-A: genetics of antidepressants; Sanger: Sanger sequencing; US: United States of America; xTAG v3: Luminex xTAG CYP2D6 Kit v3; NL: Netherlands.

a See Supplemental Table S9 for further details about sample ancestry.

b According to the FDA-approved list of CYP2D6 star alleles that AmpliChip is capable of detecting (see Table 2).

c Refers to LRS CYP2D6 genotypes that contained one or more different star alleles than their non-LRS comparison genotype.

d According to the consensus CYP2D6 genotype to phenotype translation method (Caudle et al., 2020).

e Single study with three independent cohorts, with samples belonging to each cohort specified.

The outcomes data of the included studies are also presented in Table 3. The percentage of LRS CYP2D6 genotypes that were AmpliChip-undetectable ranged from 1.8% to 94.1% across studies, or 1.2% to 88.2% when only counting those with a non-normal metaboliser phenotype. The percentage of LRS CYP2D6 genotypes that were discordant with non-LRS genotypes (i.e. contained different star alleles) ranged from 4% to 82.4% across studies, or 4% to 76.5% when only counting those with a different metaboliser phenotype.

### Quality assessment of included studies

In the context of this review, all studies were found to be at risk of bias in one or more domains of the Quality Assessment of Diagnostic Accuracy Studies-2 (QUADAS-2) or the Quality Assessment of Diagnostic Accuracy Studies-Comparative (QUADAS-C; see Supplemental Table S10). A common concern (*k* = 5) was the absence of reference testing (e.g. Sanger sequencing), or the use of such testing with only a subset of samples, to validate the findings of LRS or non-LRS tests. It has been argued, however, that LRS may be considered a gold standard genotyping technology (Kovaka et al., 2023). Another common concern (*k* = 5) was a lack of information regarding sampling methods, as certain methods may introduce more bias than others (e.g. selective versus random sampling). Other issues included using LRS with knowledge of reference testing results (*k* = 1), using referencing testing with knowledge of LRS or non-LRS test results (*k* = 3) and using LRS with knowledge of non-LRS test results (*k* = 2).

Two studies were deemed to have applicability concerns (i.e. possible incompatibility with review objectives) in one or more domains of the QUADAS-2 (see Supplemental Table S10). In these studies, LRS was used selectively for samples with CYP2D6 genotypes deemed to be novel (Fukunaga et al., 2021), complex (Qiao et al., 2016) or suitable for validating LRS (Qiao et al., 2016) based on prior non-LRS testing. Unlike the remainder of the studies, which were nonselective with LRS testing, these two studies were not considered to represent the general population of individuals who undergo CYP2D6 testing. Hence, to avoid overestimating the improvements offered by LRS, Fukunaga et al. (2021) and Qiao et al. (2016) were excluded from the meta-analysis of improvement in CYP2D6 phenotyping sensitivity (Figure 3). For reference, a meta-analysis that included these two studies is shown in Supplemental Figure S1. As these studies conducted both LRS and non-LRS genotyping, there were also concerns that they might overestimate the improvements in CYP2D6 phenotyping accuracy offered by LRS. However, the sensitivity and accuracy improvements demonstrated by LRS were still examined in these studies, albeit separately, to explore the potential benefits of LRS when used selectively (e.g. to resolve complex CYP2D6 genotypes).

### Summary and quality appraisal of findings

A summary and quality appraisal of review findings is presented in Table 4. Across the review analyses, LRS demonstrated an improvement in CYP2D6 phenotyping sensitivity (IPS) in 2%–88% of individuals and an improvement in CYP2D6 phenotyping accuracy (IPA) in 4%–76% of individuals. As mentioned in the quality assessment of included studies, all studies were found to be at risk of bias in one or more domains, and hence no review findings were deemed to be of ‘high’ quality. Findings were deemed to be of ‘moderate’ quality if they were characterised by precision (i.e. large sample size and narrow positive confidence interval) and consistency (i.e. low or no heterogeneity). Findings were deemed to be of ‘low’ quality if they were characterised by inconsistency (i.e. substantial heterogeneity) or imprecision (i.e. small sample size, wide confidence interval or confidence interval including zero). However, in the meta-analysis of IPS, findings were deemed to be of ‘moderate’ rather than ‘low’ quality, as their imprecision and inconsistency were explained by differences across geographic regions during subgroup analysis. These differences are consistent with observations that CYP2D6 allele frequencies vary significantly across global populations (Gaedigk et al., 2017).

**Table 4.** Summary and quality appraisal of findings.

Outcome	Analysis	*n* (*k*)	Effect size (95% CI)	Heterogeneity (*I*^2^)	Quality of finding
Improvement in CYP2D6 phenotyping sensitivity (IPS) demonstrated by LRS	Europe and Oceania combined (meta-analysis)	1385 (4)	10% (2, 18)	96%	Moderate^a^
	Europe (subgroup analysis)	803 (1)^b^	2% (1, 4)	22% (NS)	Moderate^c^
	Oceania (subgroup analysis)	582 (3)	20% (17, 23)	0% (NS)	Moderate^c^
	Selective^d^: genotypes deemed novel	17 (1)	88% (72, 100)	None^e^	Low^f^
	Selective^d^: genotypes deemed complex or suitable for validating LRS	9 (1)	33% (1, 66)	None^e^	Low^f^
Improvement in CYP2D6 phenotyping accuracy (IPA) demonstrated by LRS	LRS versus AmpliChip	567 (1)^g^	4% (2, 7)	None^e^	Moderate^c^
	LRS versus Sanger sequencing	25 (1)	4% (−6, 14)	None^e^	Low^f^
	Selective^d^: LRS versus MiSeq for genotypes deemed novel	17 (1)	76% (55, 98)	None^e^	Low^f^
	Selective^d^: LRS versus xTAG v3 for genotypes deemed complex or suitable for validating LRS	9 (1)	33% (1, 66)	None^e^	Low^f^

*n*: Clinical samples genotyped with LRS; *k*: number of studies; CI: confidence interval; LRS: long-read sequencing; NS: nonsignificant; AmpliChip: Roche AmpliChip CYP450; MiSeq: Illumina MiSeq; xTAG v3: Luminex xTAG CYP2D6 Kit v3.

No findings were deemed to be of high quality, as all included studies were found to be at risk of bias in one or more domains.

a Moderate due to large sample size, and differences across geographic regions (subgroup analysis) explaining the substantial heterogeneity and width of confidence interval.

b Single study with three independent cohorts (van der Lee et al., 2021).

c Moderate due to large sample size, narrow positive confidence interval and low or no heterogeneity.

d LRS was used selectively for samples with CYP2D6 genotypes deemed to be novel (Fukunaga et al., 2021), complex or suitable for validating LRS (Qiao et al., 2016) based on prior non-LRS testing.

e No heterogeneity as the finding was based on a single study or cohort (*I*^2^ figure not provided).

f Low due to imprecision, as evidenced by small sample size, wide confidence interval or confidence interval including zero.

g CYPTAM cohort of the van der Lee et al. (2021) study.

### Improvement in phenotyping sensitivity: LRS versus CYP2D6 genotypes detectable with AmpliChip

#### Descriptive data

**Figure 2.**
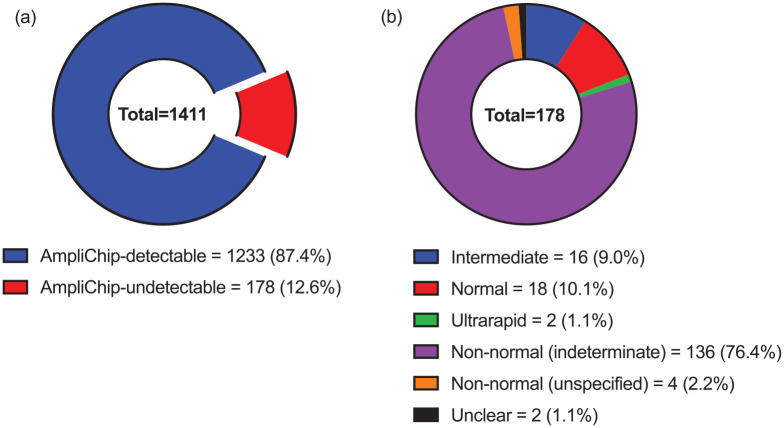
LRS CYP2D6 genotypes categorised according to (a) detectability with AmpliChip and (b) metaboliser phenotype when undetectable with AmpliChip. Non-normal (indeterminate): LRS-derived metaboliser phenotypes that would be categorised as ‘indeterminate’; non-normal (unspecified): LRS genotypes containing a star allele reported only as ‘hybrid’, which would predict a poor, intermediate or indeterminate metaboliser phenotype, depending on the specific hybrid star allele; Unclear: LRS genotypes containing a star allele reported only as ‘conversion’, which would predict a normal or non-normal metaboliser, depending on the specific conversion star allele. Detectability with AmpliChip was determined using the FDA-approved list of detectable star alleles (see Table 2), and metaboliser phenotypes were determined using the consensus CYP2D6 genotype to phenotype translation method (Caudle et al., 2020). LRS: long-read sequencing.

An overview of LRS CYP2D6 genotypes according to their detectability with AmpliChip is provided in Figure 2. Most of the 1411 LRS genotypes were AmpliChip-detectable (87.4%) and the remainder were AmpliChip-undetectable (12.6%). Of the 178 AmpliChip-undetectable genotypes, 158 (88.8%) predicted non-normal metaboliser phenotypes, including intermediate (9%), ultrarapid (1.1%), indeterminate (76.4%) and unspecified non-normal (2.2%) phenotypes. Most of the AmpliChip-undetectable genotypes predicted indeterminate metaboliser phenotypes (136; 76.4%), as they were based on CYP2D6 alleles of uncertain or unknown function (see Supplemental Table S4). However, 96 (70.6%) of the 136 indeterminate phenotypes were based on CYP2D6 alleles with preliminary functional data, of which 90 (93.8%) were based on alleles that had exhibited subnormal function (see Supplemental Table S7). Approximately 112 (82.4%), 12 (8.8%) and 8 (5.9%) of the indeterminate phenotypes were found in those of Oceanian, Japanese and European ancestry, respectively (see Supplemental Tables S5 and S9). Study-level data for AmpliChip-undetectable LRS CYP2D6 genotypes are shown in Supplemental Table S5.

#### Examination of improvement in phenotyping sensitivity

**Figure 3.**
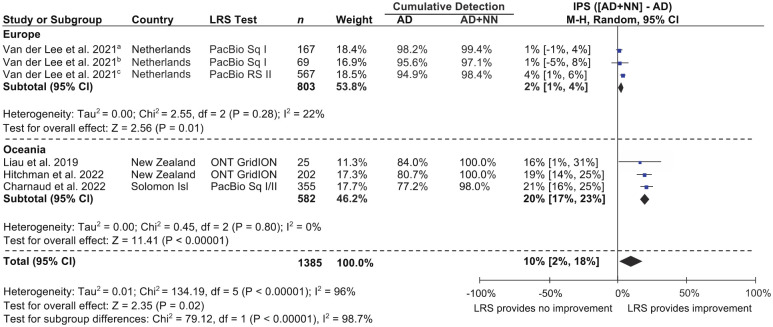
Improvement in CYP2D6 phenotyping sensitivity (IPS) demonstrated by LRS. Detectability with AmpliChip was determined using the FDA-approved list of detectable star alleles (see Table 2), and metaboliser phenotypes were determined using the consensus CYP2D6 genotype to phenotype translation method (Caudle et al., 2020). Non-normal metaboliser phenotypes included those that would be categorised as ‘poor’, ‘intermediate’, ‘ultrarapid’ or ‘indeterminate’ using the consensus CYP2D6 genotype to phenotype translation method. *n*: Clinical samples genotyped with LRS; AD: percentage of CYP2D6 genotypes detected by LRS that were AmpliChip-detectable; NN: percentage of CYP2D6 genotypes detected by LRS that were AmpliChip-undetectable and predicted a non-normal metaboliser phenotype; PacBio: Pacific Biosciences; Sq: sequel; ONT: Oxford Nanopore Technology. ^a^Independent study cohort 2: Breast cancer patients receiving tamoxifen (CYPTAM-BRUT cohort). ^b^Independent study cohort 3: Hospital patients receiving venlafaxine (Venlafaxine cohort). ^c^Independent study cohort 1: Breast cancer patients receiving tamoxifen (CYPTAM cohort).

A meta-analysis of the improvement in CYP2D6 phenotyping sensitivity (IPS) demonstrated by LRS is shown in Figure 3. IPS referred to the percentage of LRS CYP2D6 genotypes in each study or cohort that were AmpliChip-undetectable and predicted a non-normal metaboliser phenotype. As mentioned in the quality assessment of included studies, two studies (Fukunaga et al., 2021; Qiao et al., 2016) were excluded from this meta-analysis due to using LRS selectively and may have otherwise exaggerated the IPS (see Supplemental Figure S1). Across the remaining four studies or their cohorts, IPS ranged from 1 to 21% (see Figure 3). Within the European subgroup, which was represented by a single study with three independent cohorts, IPS ranged from 1% to 4%. Within the Oceanian subgroup, which was represented by three independent studies, IPS ranged from 16% to 21%. Combined IPS was 2% for the European subgroup, 20% for the Oceanian subgroup and 10% overall. All IPS values were statistically significant, including at the cohort, study, subgroup and overall level, aside from two of three cohorts in the European subgroup. Heterogeneity within subgroups was low and nonsignificant. Heterogeneity between subgroups, and at the overall level, was high and significant.

The improvement in CYP2D6 phenotyping sensitivity (IPS) demonstrated by LRS was considered separately for the two studies excluded from the meta-analysis (Fukunaga et al., 2021; Qiao et al., 2016). In the study by Fukunaga et al. (2021), which used LRS for suspected novel CYP2D6 genotypes, IPS was 88% (95% CI = (72, 100); Country = Japan; LRS test = Pacific Biosciences Sequel I; *n* = 17). In the study by Qiao et al. (2016), which used LRS for CYP2D6 genotypes deemed complex or suitable for validating LRS, IPS was 33% (95% CI = (1, 66); Country = USA; LRS test = Pacific Biosciences RSII; *n* = 9).

When considering all six studies, an asymmetrical funnel plot was observed for IPS (see Supplemental Figure S2). However, this asymmetry was deemed to reflect the selective use of LRS in the studies by Fukunaga et al. (2021) and Qiao et al. (2016), resulting in a larger effect size than studies without selective testing. Publication bias was therefore not indicated.

### Improvement in phenotyping accuracy: LRS versus non-LRS technology

#### Descriptive data

**Figure 4.**
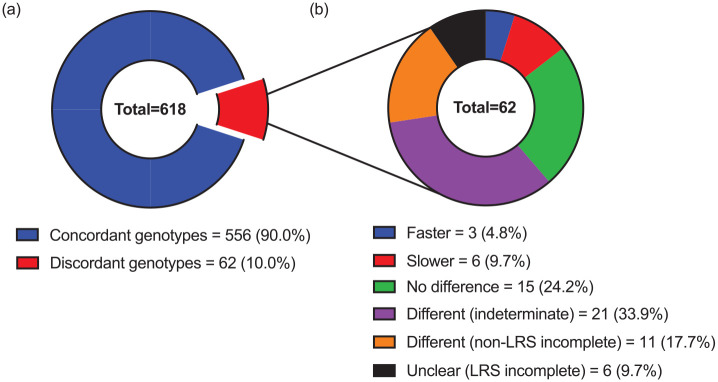
LRS CYP2D6 genotypes with comparison data categorised by (a) concordance with their non-LRS comparison genotype and (b) any difference in metaboliser phenotype when discordant. Metaboliser phenotypes are determined using the consensus CYP2D6 genotype to phenotype translation method (Caudle et al., 2020). Concordant: No different star alleles between LRS and non-LRS comparison genotype; Discordant: LRS genotype contained or more different star alleles from its non-LRS comparison genotype; Faster: LRS genotype predicted faster metaboliser category (e.g. normal metaboliser instead of intermediate metaboliser); Slower: LRS genotype predicted slower metaboliser category (e.g. intermediate metaboliser instead of normal metaboliser); Different (indeterminate): LRS genotype, but not non-LRS genotype, predicted ‘indeterminate’ metaboliser phenotype; Different (non-LRS incomplete): non-LRS genotype was incomplete (i.e. one or more star alleles not specified), precluding prediction of metaboliser phenotype; Unclear (LRS incomplete): LRS genotype was incomplete (i.e. one or more star alleles not specified), precluding prediction of metaboliser phenotype.

A total of 618 (43.8%) of the 1411 clinical samples genotyped with LRS were also genotyped with non-LRS technology (i.e. genotype comparison data were available). Concordance between the respective LRS and non-LRS genotype pairs (i.e. whether they were comprised of the same star alleles) is overviewed in Figure 4. Most of the 618 LRS genotypes in question were concordant with their non-LRS comparison genotype (90%), whilst a minority were discordant (10%). Of the 62 discordant LRS genotypes, 41 (66.1%) predicted different metaboliser phenotypes than their comparison genotypes, including faster (4.8%), slower (9.7%), indeterminate (33.9%) or merely available phenotypes (17.7%; where non-LRS phenotypes were unavailable due to incomplete data). Study-level data for discordant LRS CYP2D6 genotypes are shown in Supplemental Table S8.

#### Examination of improvement in phenotyping accuracy

The improvement in CYP2D6 phenotyping accuracy (IPA) demonstrated by LRS was examined in the four studies that contained samples genotyped with both LRS and non-LRS technology. IPA referred to the percentage of LRS CYP2D6 genotypes in each study or cohort that predicted a different metaboliser phenotype than their non-LRS comparison genotype. In the study by Liau et al. (2019), IPA was 4% (95% CI = (−6, 14); Country = New Zealand; LRS test = Oxford Nanopore Technology GridION; non-LRS test = Sanger sequencing; *n* = 25). In the CYPTAM cohort of the study by van der Lee et al. (2021), IPA was also 4% (95% CI = (2, 7); Country = Netherlands; LRS test = Pacific Biosciences RS II; non-LRS test = Roche AmpliChip CYP450; *n* = 567).

As mentioned in the quality assessment of the included studies, the other two studies with both LRS and non-LRS data (Fukunaga et al., 2021; Qiao et al., 2016) used LRS selectively, which may have exaggerated the IPA. In the study by Fukunaga et al. (2021), which used LRS for suspected novel CYP2D6 genotypes, IPA was 76% (95% CI = (55, 98); Country = Japan; LRS test = Pacific Biosciences Sequel I; non-LRS test = Illumina MiSeq; *n* = 17). In the study by Qiao et al. (2016), which used LRS for CYP2D6 genotypes deemed complex or suitable for validating LRS, IPA was 33% (95% CI = (1, 66); Country = USA; LRS test = Pacific Biosciences RSII; non-LRS test = Luminex xTAG CYP2D6 Kit v3; *n* = 9).

When considering all four studies with both LRS and non-LRS data, an asymmetrical funnel plot was observed for IPA (see Supplemental Figure S3). However, this asymmetry was deemed to reflect the selective use of LRS in the studies by Fukunaga et al. (2021) and Qiao et al. (2016), resulting in a larger effect size than studies without selective testing. Publication bias was therefore not indicated.

## Discussion

### Summary and interpretation of findings

In this novel systematic review, LRS was found to offer improvements in CYP2D6 phenotyping sensitivity (IPS) and accuracy (IPA) compared to established genetic tests. IPS, or the percentage of LRS CYP2D6 metaboliser phenotypes that were non-normal and would be undetectable with the FDA-approved AmpliChip test, was 10% in a meta-analysis of four studies (*n* = 1385). Subgroup analysis indicated a higher figure amongst Oceanians (20%; *n* = 582) and a lower figure amongst Europeans (2%; *n* = 803). IPA, or the percentage of LRS CYP2D6 metaboliser phenotypes that were mischaracterised by non-LRS testing, was low when LRS was compared to AmpliChip in a large European cohort (4%; *n* = 567). IPA was low and nonsignificant when LRS was compared to Sanger sequencing in a small Oceanian study (4%; *n* = 25). In two studies that used selective testing (i.e. non-LRS pretesting to determine eligibility for LRS testing), IPS and IPA were considered separately and found to be markedly higher. One of these studies used LRS and the FDA-approved xTAG CYP2D6 test for genotypes deemed to be complex or suitable for validating LRS (IPS = 33%; IPA = 33%; *n* = 9). The other study used LRS and SRS for genotypes suspected of being novel in a Japanese population (IPS = 88%; IPA = 76%; *n* = 17). Thus, LRS appears to detect CYP2D6 phenotypes that are missed or miscategorised by established genetic tests, the extent to which varies considerably by population.

This review builds upon previous research to show that the more accurate CYP2D6 genotyping offered by LRS (Buermans et al., 2017; Yang et al., 2017) translates into improved phenotyping. Improvements in the detection of non-normal and mischaracterised metaboliser phenotypes were both greatest when LRS was used selectively (e.g. for complex or suspected novel genotypes), particularly amongst those of Japanese ancestry. Furthermore, improvements in the detection of non-normal metaboliser phenotypes were greater amongst those of Oceanian than European ancestry. Hence, CYP2D6 genotypes in Asia and Oceania that tend to be missed by established tests (Gaedigk et al., 2017), but identified by LRS, may be clinically significant (e.g. associated with non-normal metabolism). Conversely, LRS did not appear to improve the detection of mischaracterised phenotypes amongst Oceanians, but this finding was based on a very small sample of unspecified ancestry and an expensive, low-throughput comparison test (Sanger sequencing; Hu et al., 2021), limiting its clinical relevance. In any case, the improvement in CYP2D6 phenotyping demonstrated by LRS may be clinically significant, particularly amongst non-Europeans and those with novel or complex genotypes.

Improvements in CYP2D6 phenotyping largely reflected the identification of ‘indeterminate’ metaboliser phenotypes by LRS that would be missed or had been miscategorised (e.g. as ‘normal’) by non-LRS tests. Indeterminate phenotypes are those yet to be ascribed abnormal (i.e. rapid or slow) or normal metabolism, as they are based on inadequately researched CYP2D6 alleles (Caudle et al., 2020). The lack of research into these alleles may reflect their tendency to be missed by established genetic tests (Gaedigk et al., 2017), thereby overestimating their rarity and underestimating their clinical relevance. Accordingly, the prevalence and clinical impact of indeterminate CYP2D6 metabolisers across populations has been difficult to gauge. This review found that 136 (9.6%) of the 1411 individuals genotyped with LRS were indeterminate metabolisers, of which approximately 112 (82.4%) and 12 (8.8%) were of Oceanian and Japanese ancestry, respectively. Ninety-six (70.6%) of the indeterminate phenotypes were based on CYP2D6 alleles with preliminary functional data, of which 90 (93.8%) were based on alleles that had exhibited subnormal function (i.e. slow metabolism). Hence, the prevalence and clinical impact of indeterminate CYP2D6 metaboliser phenotypes may be greater than once thought, particularly amongst non-Europeans.

### Limitations of the evidence

As a result of this review, several gaps were identified in the literature concerning LRS of CYP2D6. First, nearly all included studies failed to adequately describe their sampling method, leading to possible over- or underestimation of the benefits of LRS. For example, if individuals were recruited for CYP2D6 testing based on pharmacotherapy nonresponse, they may have been more likely to possess complex genotypes and non-normal metabolism, thereby exaggerating the benefits of LRS. Second, some studies recruited from populations inherently more likely to contain non-normal CYP2D6 metabolisers (e.g. adverse drug reaction cohorts), once again leading to possible overestimation of LRS benefits. Third, nearly all studies omitted reference testing to validate either a subset or the entirety of reported CYP2D6 genotypes. Though LRS has been posited as a gold standard genotyping technology (Kovaka et al., 2023), the failure to uniformly apply a reference test with comparable accuracy (e.g. Sanger sequencing) may limit the validity assessment of LRS findings. Finally, several studies used tests with knowledge of other test results (e.g. reference testing with knowledge of LRS genotypes), possibly biasing test interpretation. Importantly, these limitations were identified in the context of the current review’s objective and may therefore not be relevant to the included studies’ objectives.

### Limitations of this review

There are several limitations in this review. First, due to the small number of included publications, regression tests for publication bias were not completed. Second, amongst the included studies, there was limited representation of geographic regions and ancestries, with those of European and Oceanian ancestry heavily overrepresented. Third, because no more than one study represented each unique comparison between LRS and a non-LRS technology (e.g. LRS vs SRS), no meta-analyses of improvement in CYP2D6 phenotyping accuracy could be conducted. Fourth, there was no subgroup analysis for different LRS test brands or models, which may have helped determine whether certain LRS tests are more advantageous. Fifth, describing ‘indeterminate’ metaboliser phenotypes as ‘non-normal’ (alongside ‘poor’, ‘intermediate’ and ‘ultrarapid’ phenotypes) may have overestimated the improvements offered by LRS. Though the indeterminate phenotypes identified by LRS were based predominantly on CYP2D6 alleles exhibiting subnormal function in preliminary studies, they may ultimately be deemed to be of normal function. Sixth, as a purported gold standard genotyping technology (Kovaka et al., 2023), LRS was assumed to produce more accurate CYP2D6 genotypes than other technologies, and its benefits may have been overestimated to the degree that this assumption was incorrect. Finally, several review findings were deemed to be of low quality due to small sample sizes or wide confidence intervals (e.g. improvement in CYP2D6 phenotyping when LRS was used selectively). Confidence in these findings as true estimates of the improvements offered by LRS is therefore limited.

### Implications for practice and future research

Compared to established genetic tests, LRS demonstrated more accurate CYP2D6 metaboliser phenotyping and increased sensitivity to non-normal CYP2D6 metaboliser phenotypes. These findings may have serious clinical implications, as CYP2D6 metaboliser phenotype predicts the serum concentration of several psychotropic medications, with adverse effects and nonresponse related to high and low concentrations, respectively (Beunk et al., 2023; Bousman et al., 2023; Hicks et al., 2017). Established genetic tests may therefore produce suboptimal prescribing guidance, and subsequent poor outcomes (e.g. over- or underdosing of medication), insofar as they mischaracterise or fail to detect CYP2D6 phenotypes. By comparison, LRS may offer an incremental benefit in the safety and efficacy of psychotropic prescribing, insofar as it provides a meaningful improvement in CYP2D6 phenotyping. The current review suggests that this incremental benefit would arise in up to 4% of individuals with European ancestry, 20% of individuals with Oceanian ancestry, 33% of individuals with complex genotypes and 88% of individuals with novel genotypes and Japanese ancestry.

Current findings may help to address concerns about the feasibility and clinical utility of CYP2D6 testing, which have contributed to its slow implementation and uptake. Existing trials of CYP2D6 genotype-guided prescribing are based on non-LRS technology and have produced inconsistent results, raising doubts about the readiness of pharmacogenetic testing for routine clinical practice (Barlati et al., 2023). With more sensitive and accurate CYP2D6 phenotyping, LRS may offer safer, more reliable and more effective prescribing guidance in clinical pharmacogenetics trials, leading to more consistent benefits. Ultimately, this may encourage additional funding and infrastructure for pharmacogenetic testing, facilitating its clinical adoption. At present, LRS is more expensive than some non-LRS technology, ranging from 3 to 86 USD per sequenced gigabase of DNA compared to 2 to 30 USD for SRS (Espinosa et al., 2024). However, if LRS increases the clinical utility of CYP2D6 testing, with subsequent cost savings (e.g. decreased healthcare attendances due to treatment failure and adverse drug reactions), then it may prove superior from a cost–benefit perspective.

Whilst this review suggests that LRS may improve the clinical benefits of CYP2D6 testing, further research is needed to assess the nature and extent of these benefits, including LRS-guided psychotropic prescribing trials. However, such trials are yet unlikely to capture the full potential of LRS, as many of the CYP2D6 alleles it detects tend to be missed by established tests (Gaedigk et al., 2017) and therefore understudied. In turn, many LRS CYP2D6 genotypes are deemed to be of uncertain function (i.e. ‘indeterminate’ metabolisers), and hence omitted from genotype-based prescribing guidelines (e.g. Bousman et al., 2023). Current findings, and expert consensus (Caudle et al., 2020), suggest that many of these genotypes should be ascribed a slow (i.e. ‘intermediate’ or ‘poor’) metaboliser phenotype. However, further research is needed to confirm their function and incorporate them into prescribing guidelines. Further research is also needed to determine the benefits of CYP2D6 testing with LRS across major ethnic groups, as existing studies focus largely on Oceanian and European populations.

## Conclusion

In this review, LRS of CYP2D6 was associated with improved phenotyping sensitivity and accuracy compared to established genetic tests. Improvements were higher amongst those with Oceanian ancestry, Japanese ancestry, novel genotypes and complex genotypes, and lower amongst those of European ancestry. CYP2D6 genotyping with LRS may therefore assist with optimal selection and dosing of antidepressants, antipsychotics and other CYP2D6 substrates, particularly amongst non-Europeans and those with difficult genotypes. Clinical trials are needed to assess the benefits of CYP2D6 genotype-guided prescribing with LRS, including with respect to treatment efficacy, harms and cost–benefit analysis. However, many CYP2D6 genotypes detected with LRS tend to be missed by established tests and have therefore been understudied, resulting in unclear metaboliser phenotypes and omission from prescribing guidelines. Hence, further research is needed to determine the function of understudied CYP2D6 genotypes before the clinical utility of LRS can be fully assessed.

## Supplemental Material

sj-docx-1-jop-10.1177_02698811241268899 – Supplemental material for Long-read sequencing of CYP2D6 may improve psychotropic prescribing and treatment outcomes: A systematic review and meta-analysis Supplemental material, sj-docx-1-jop-10.1177_02698811241268899 for Long-read sequencing of CYP2D6 may improve psychotropic prescribing and treatment outcomes: A systematic review and meta-analysis by Dean Kaptsis, Martin Lewis, Michael Sorich and Malcolm Battersby in Journal of Psychopharmacology
