# Supplementary material for: Long-read sequencing of CYP2D6 may improve psychotropic prescribing and treatment outcomes: A systematic review and meta-analysis
Source: J Psychopharmacol. 2024 Sep 11;38(9):771–83. doi: 10.1177/02698811241268899 (PMC11447996; doi:10.1177/02698811241268899)
Supplement: sj-docx-1-jop-10.1177_02698811241268899 – Supplemental material for Long-read sequencing of CYP2D6 may improve psychotropic prescribing and treatment outcomes: A systematic review and meta-analysis [file sj-docx-1-jop-10.1177_02698811241268899.docx]

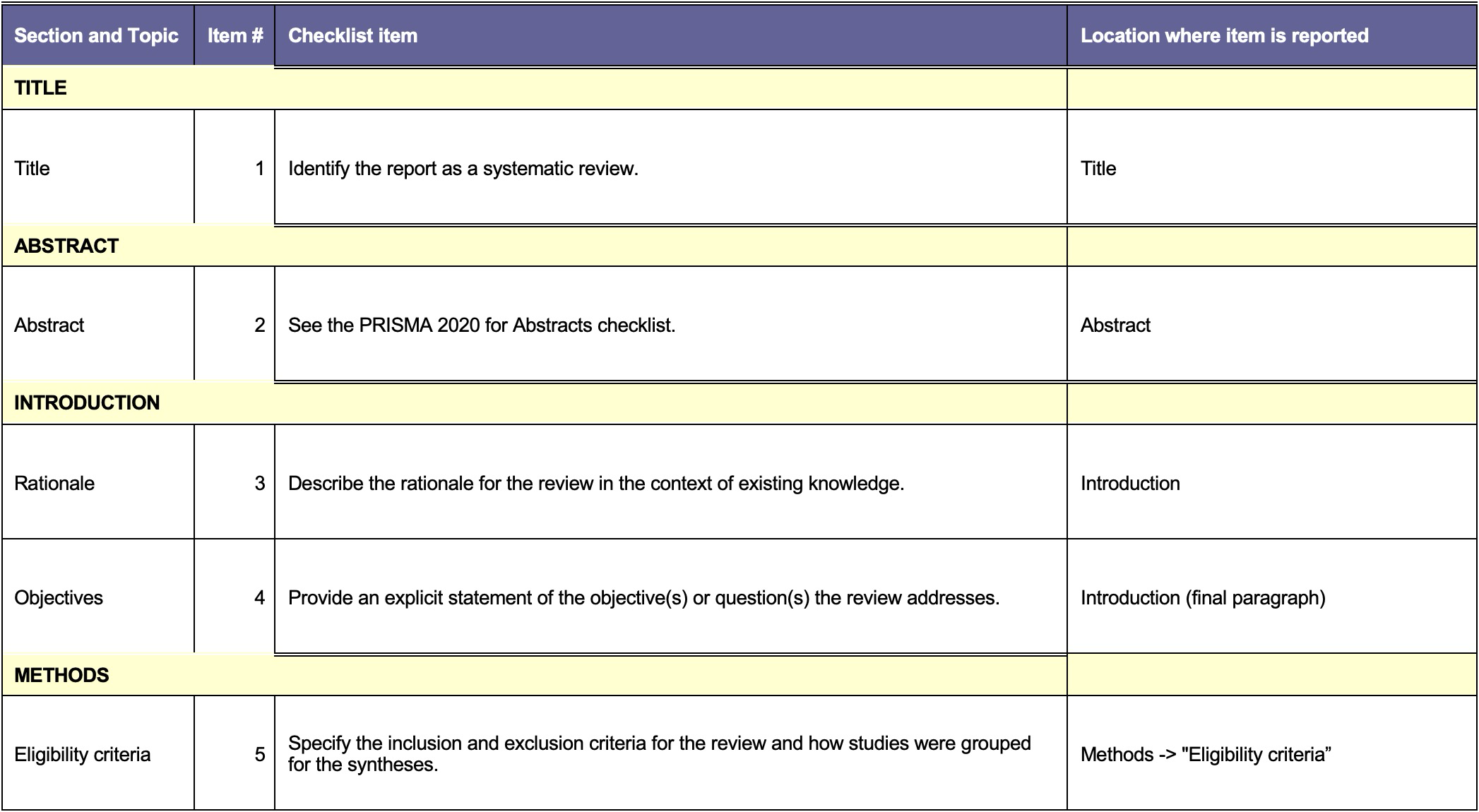


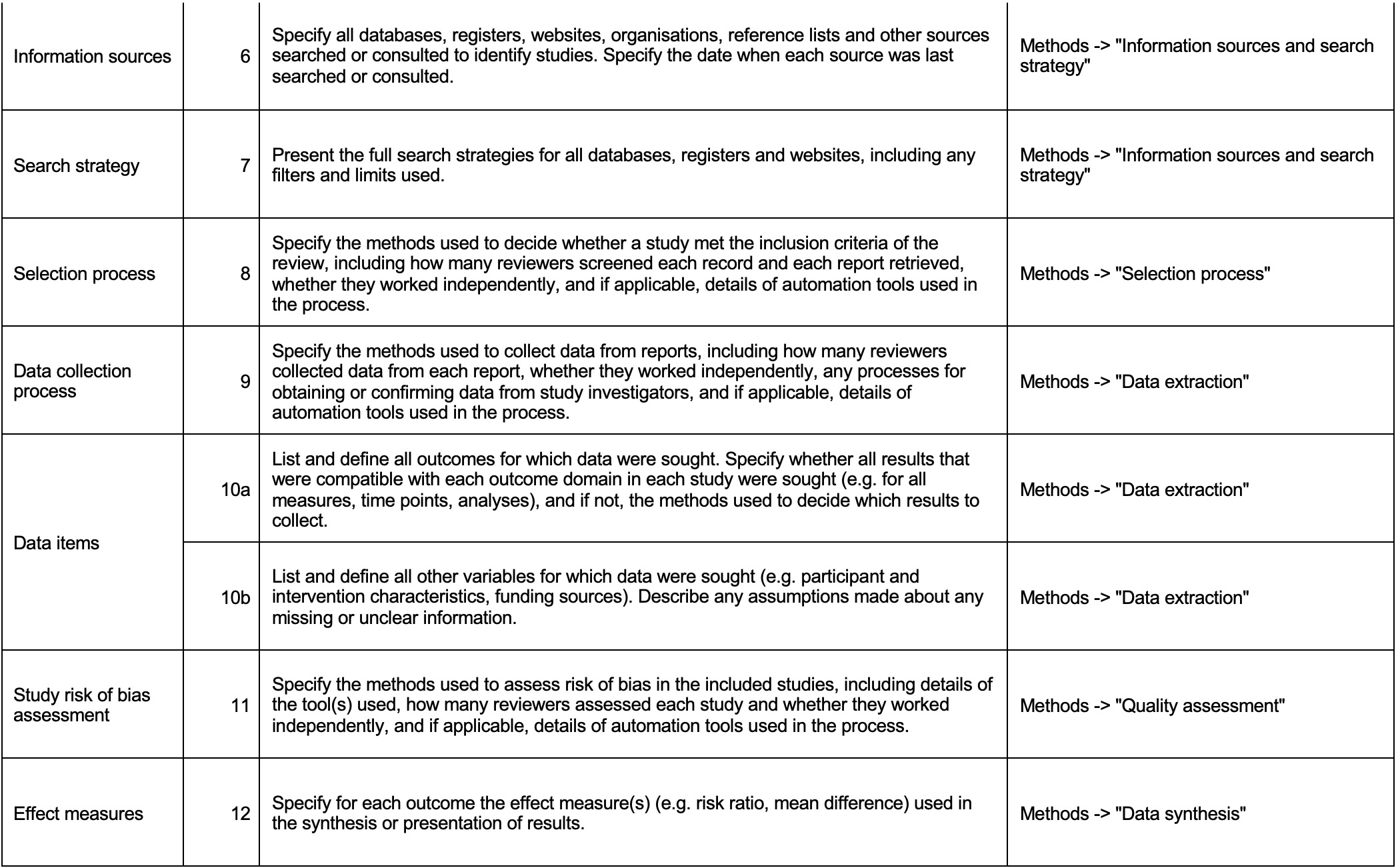


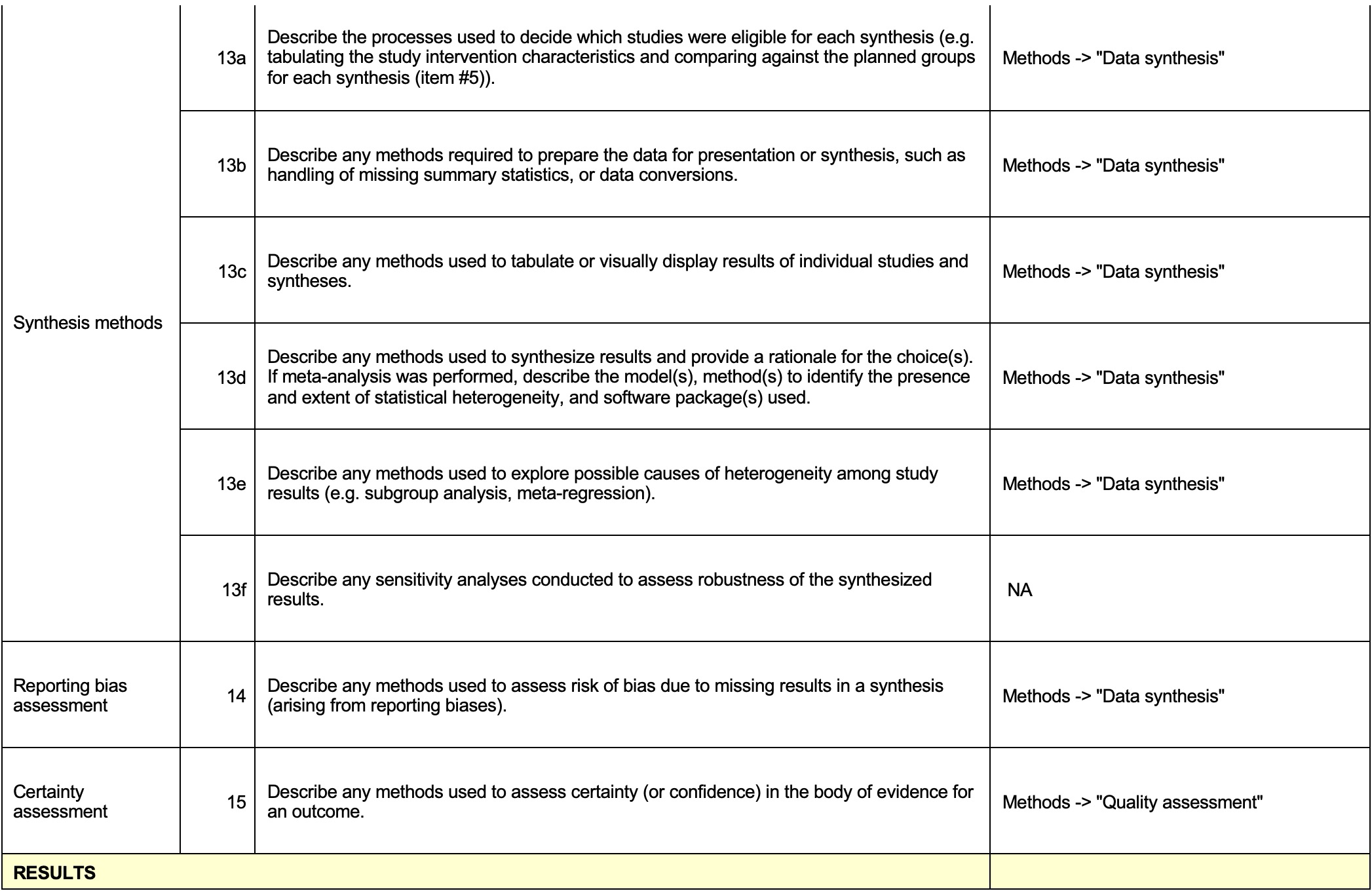


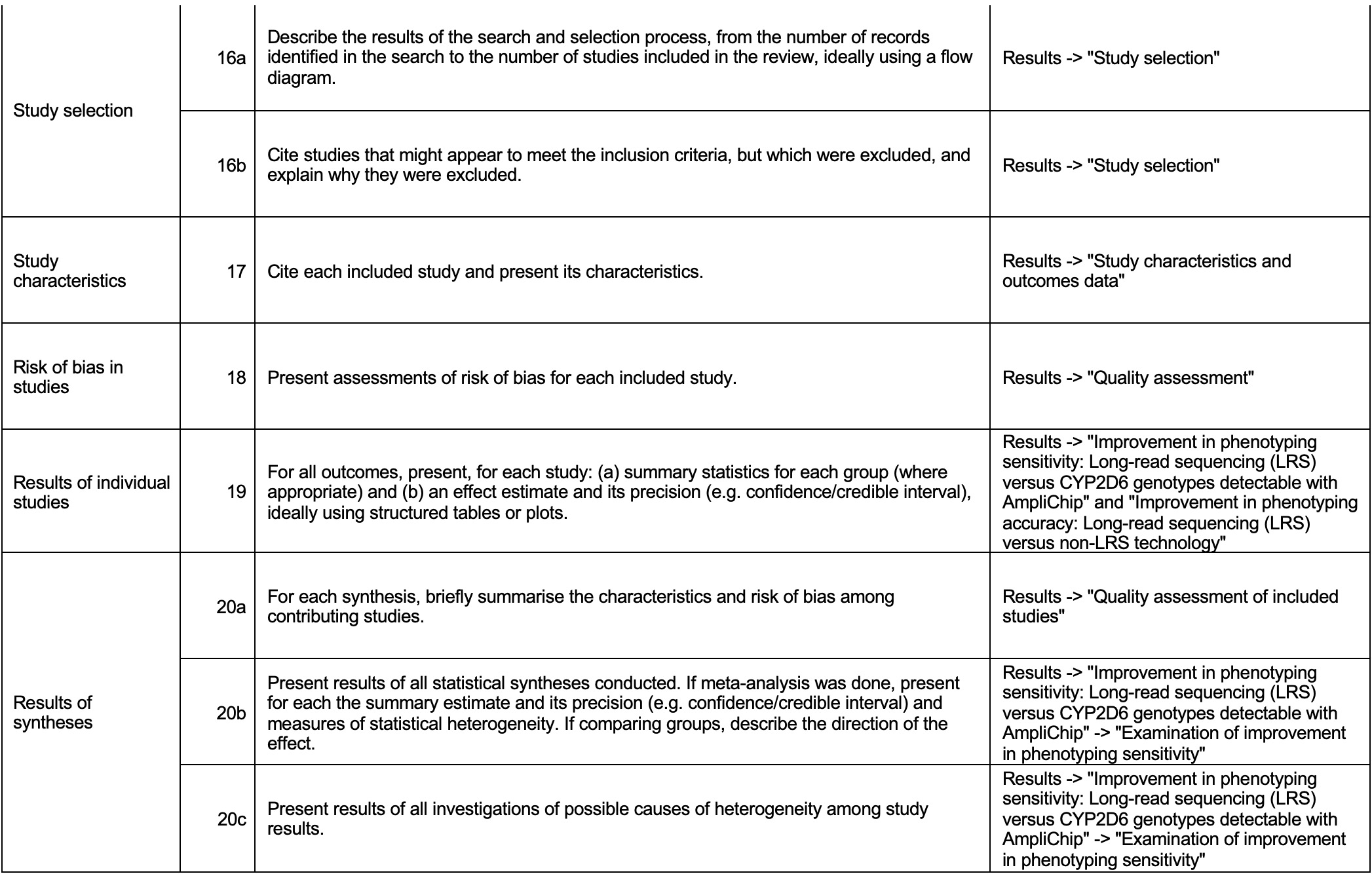


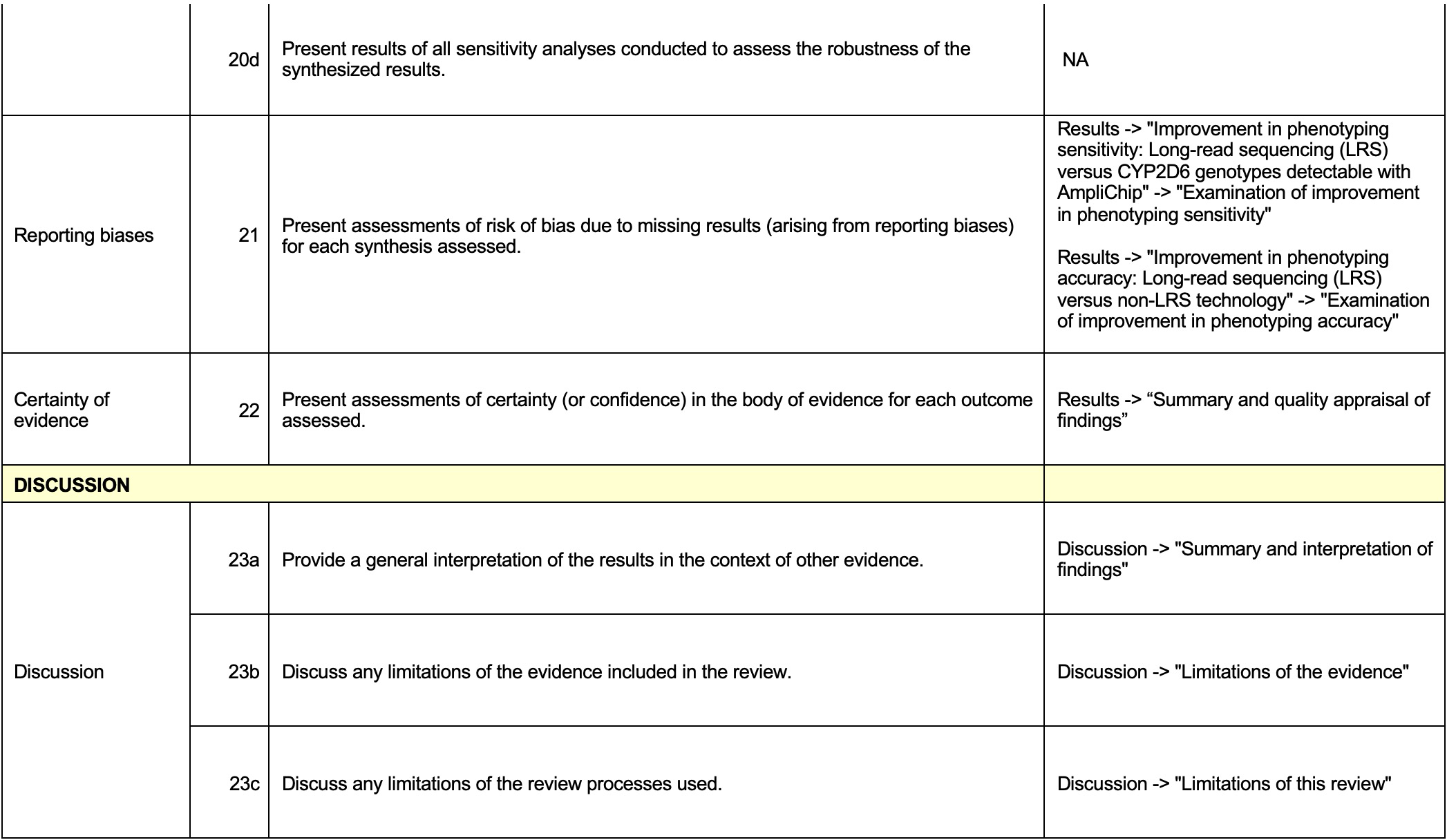


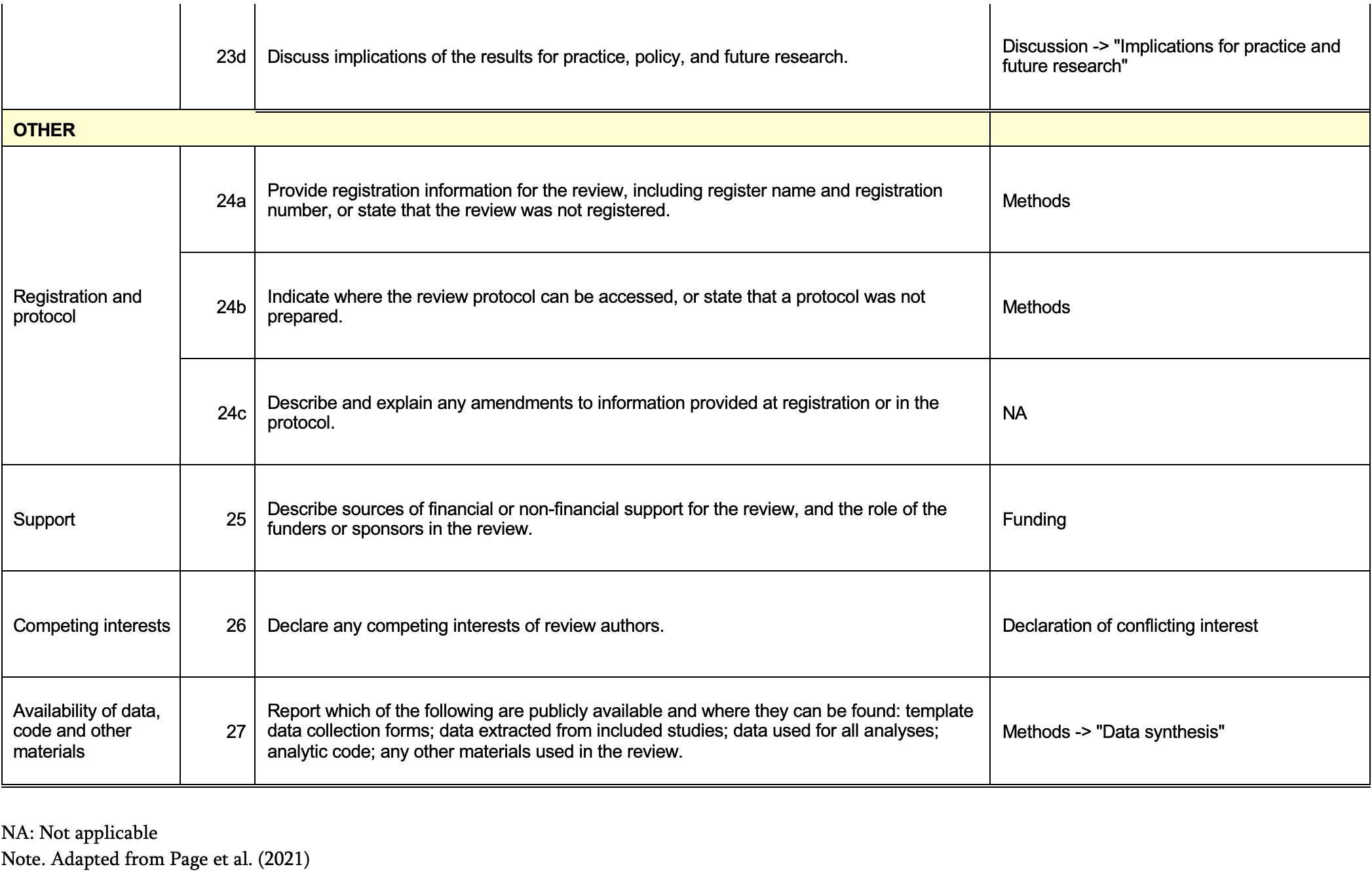

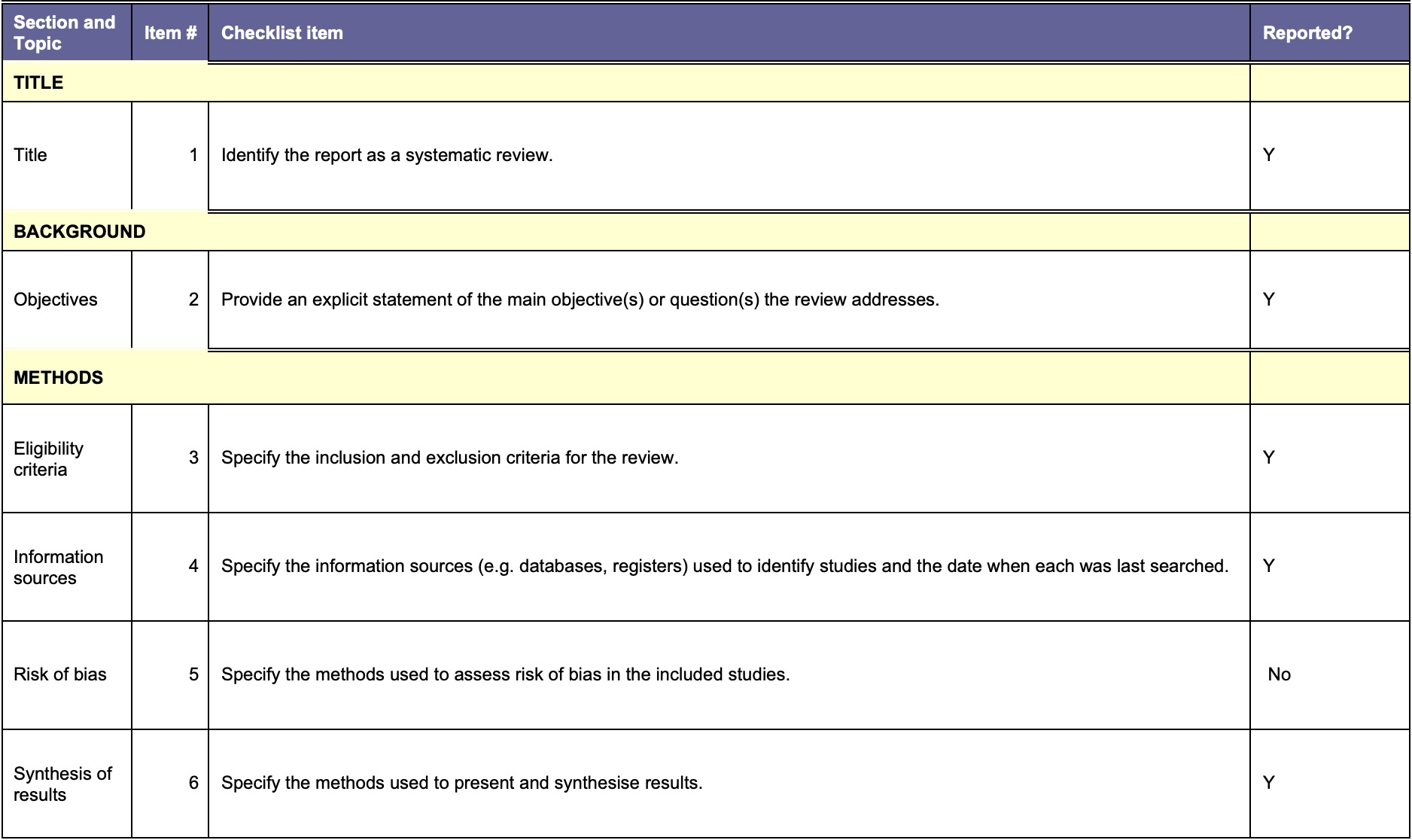


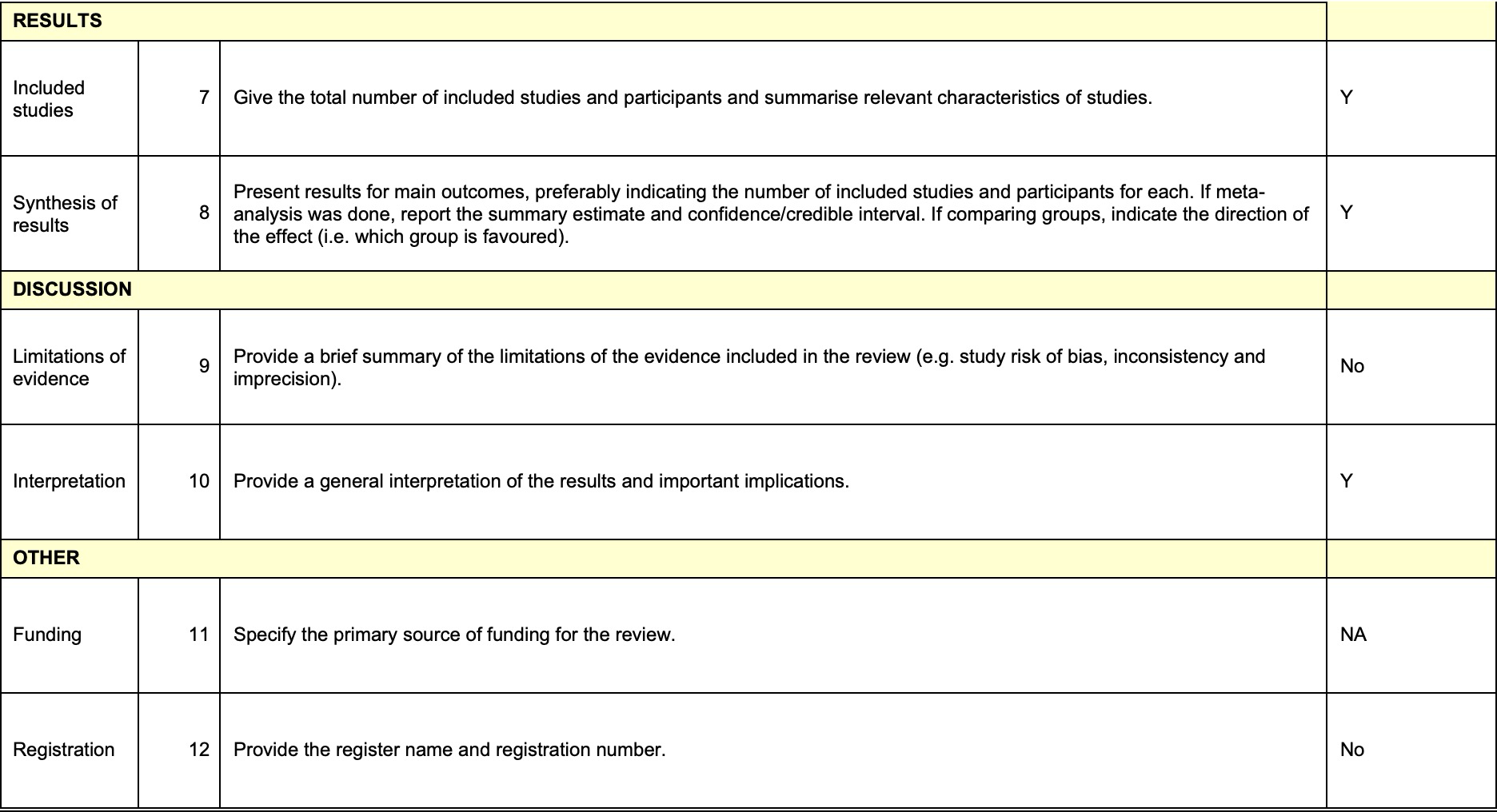

**
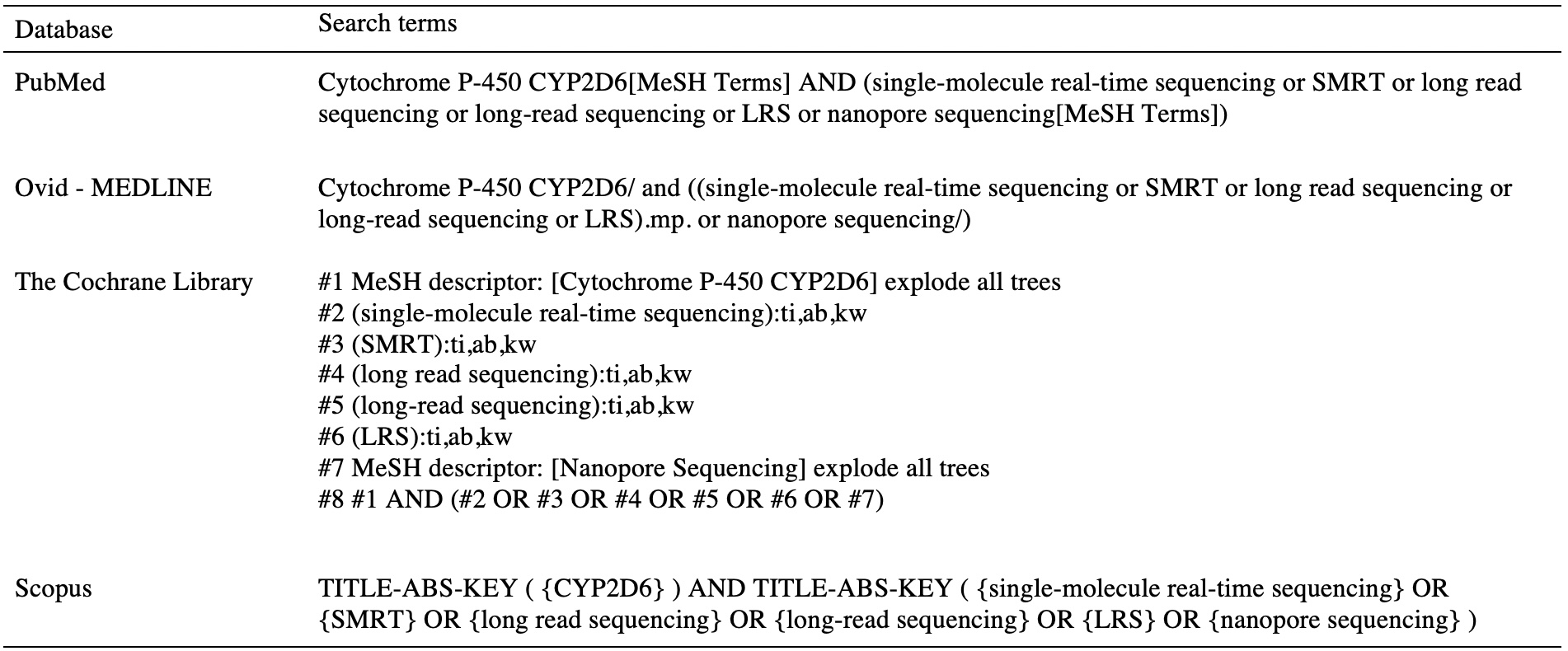
**

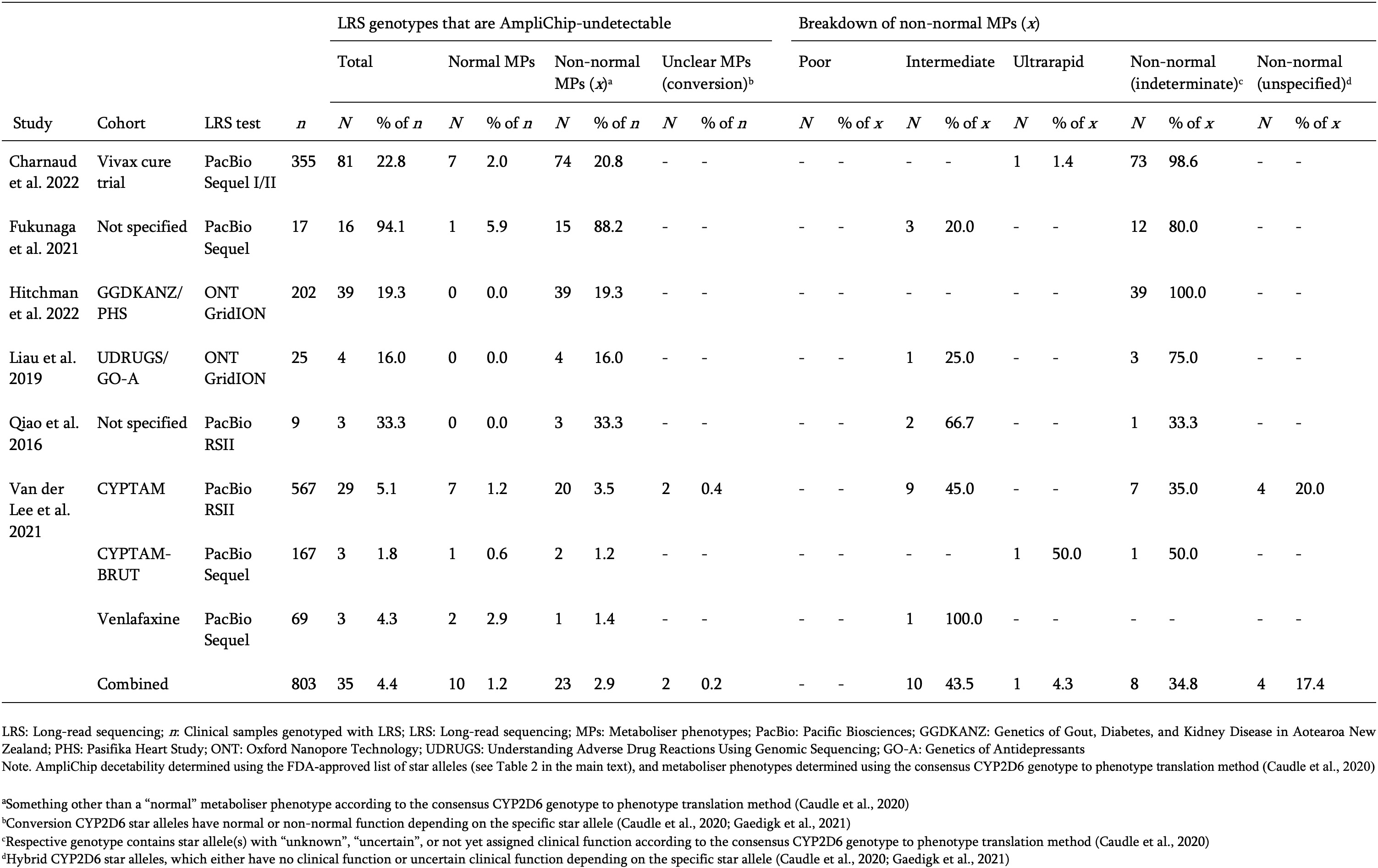

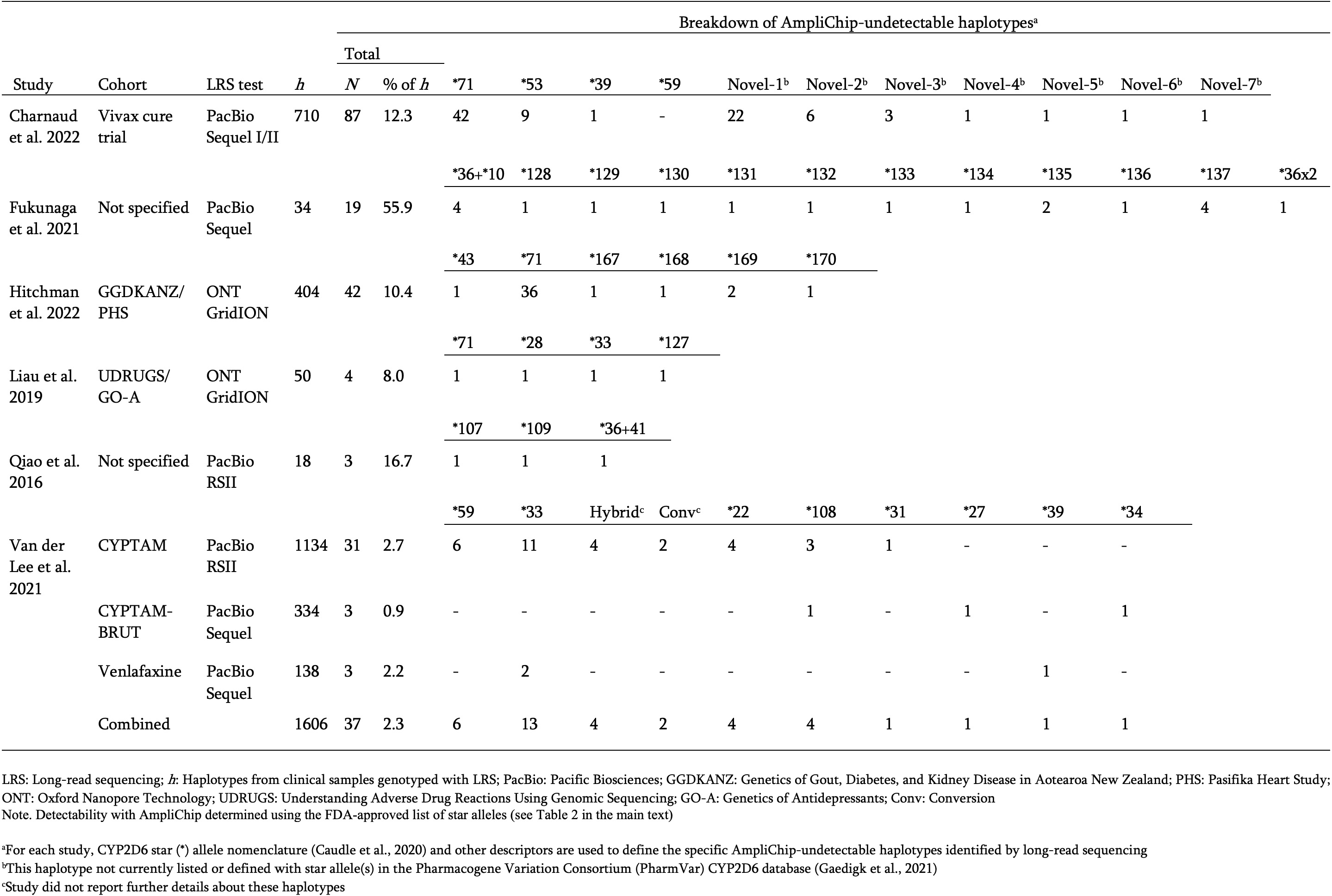

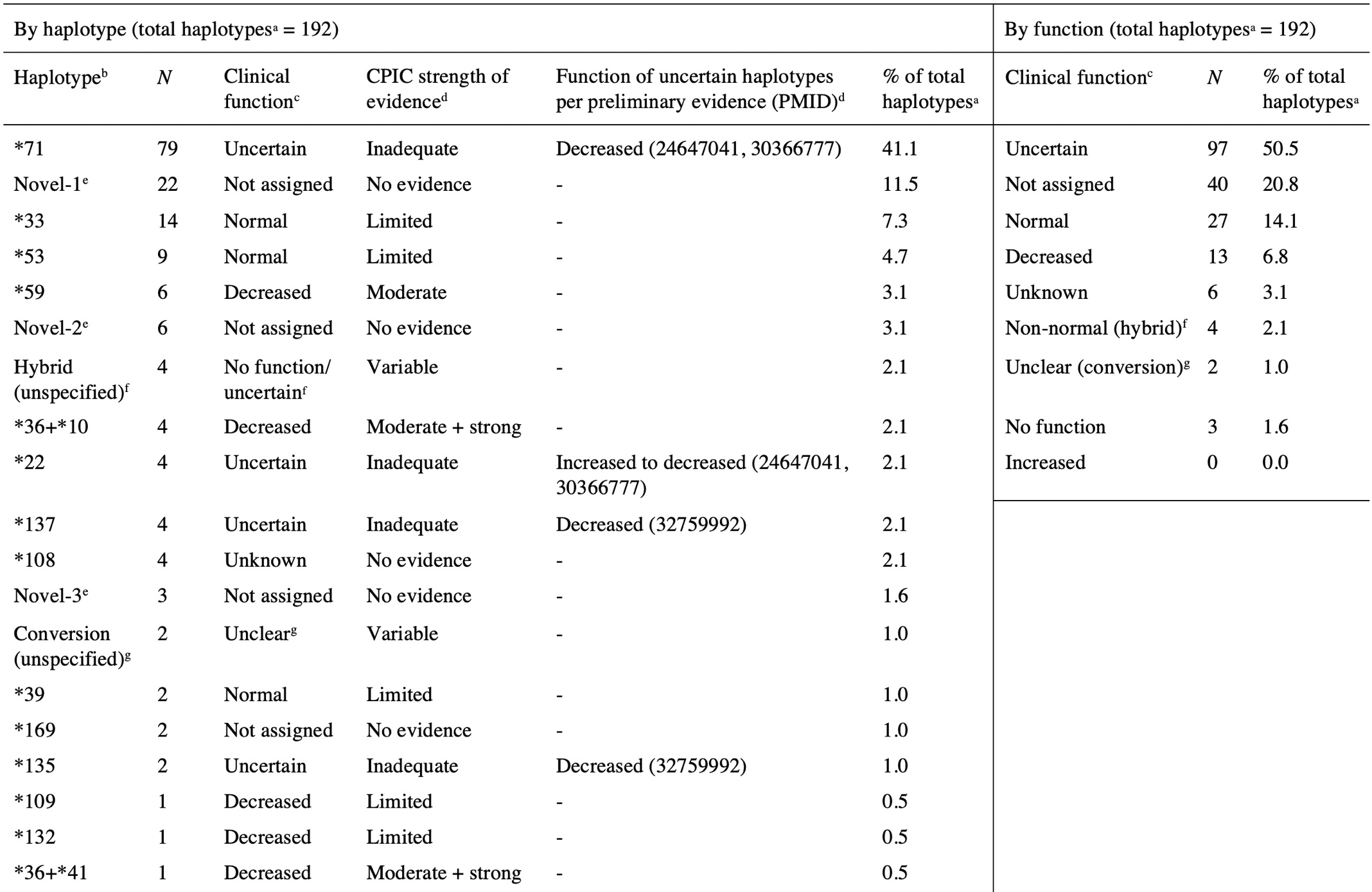


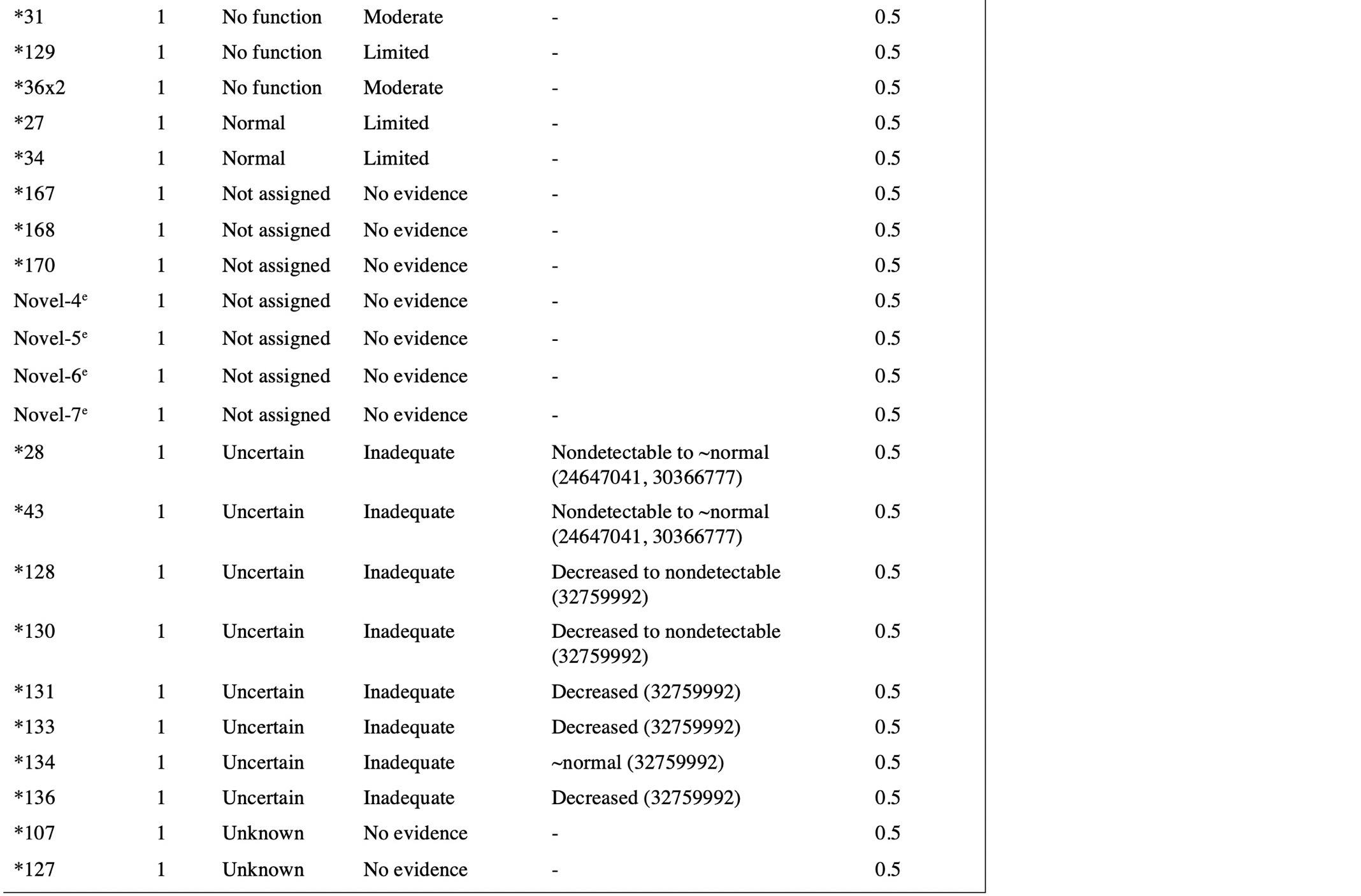


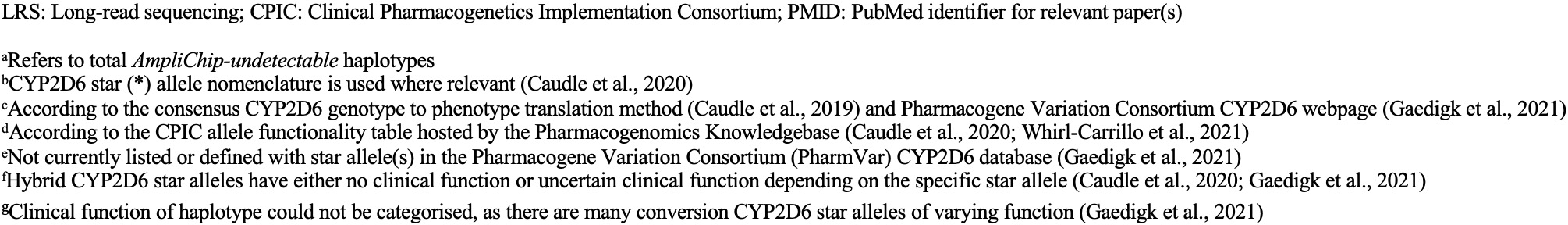

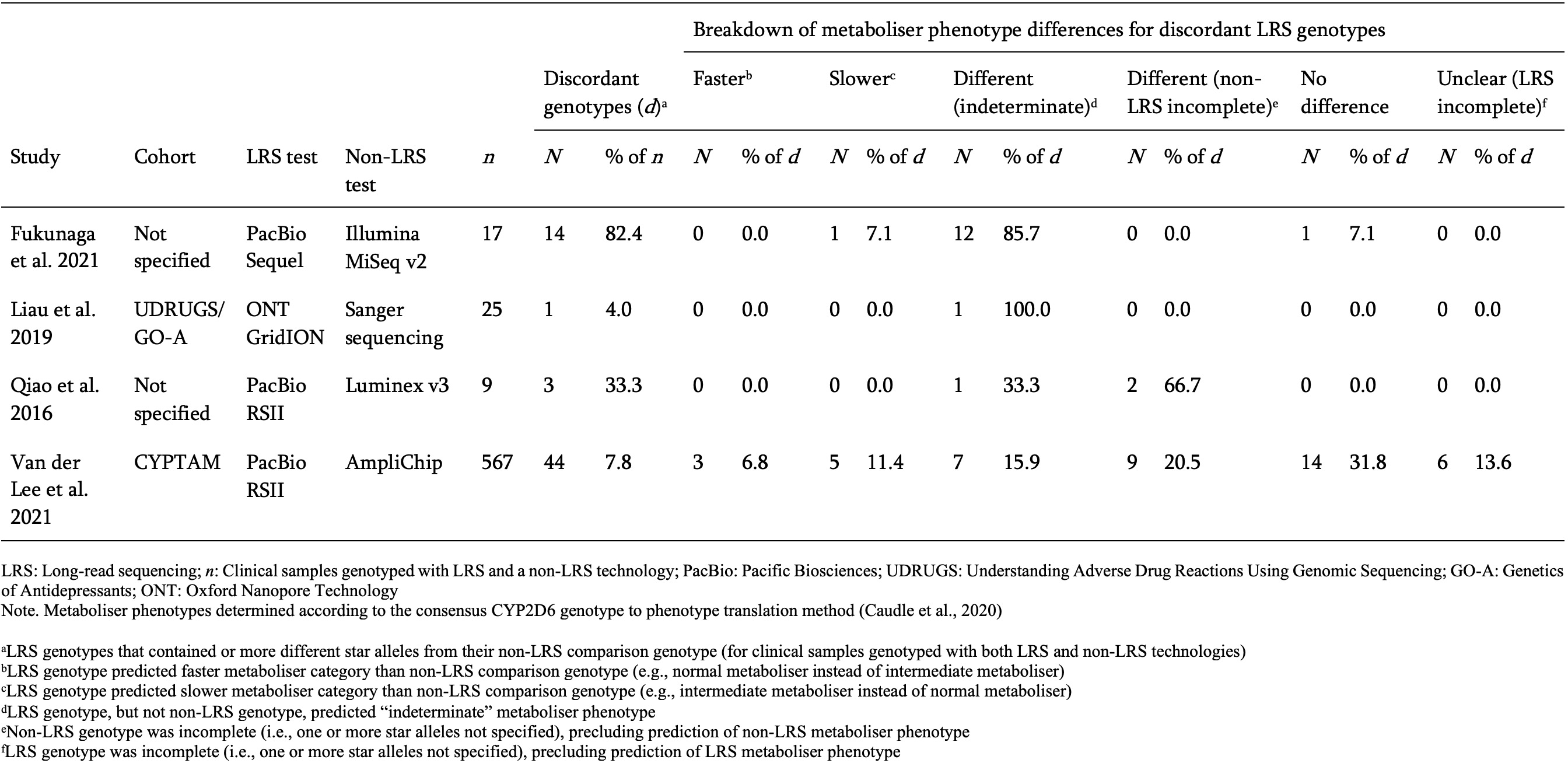

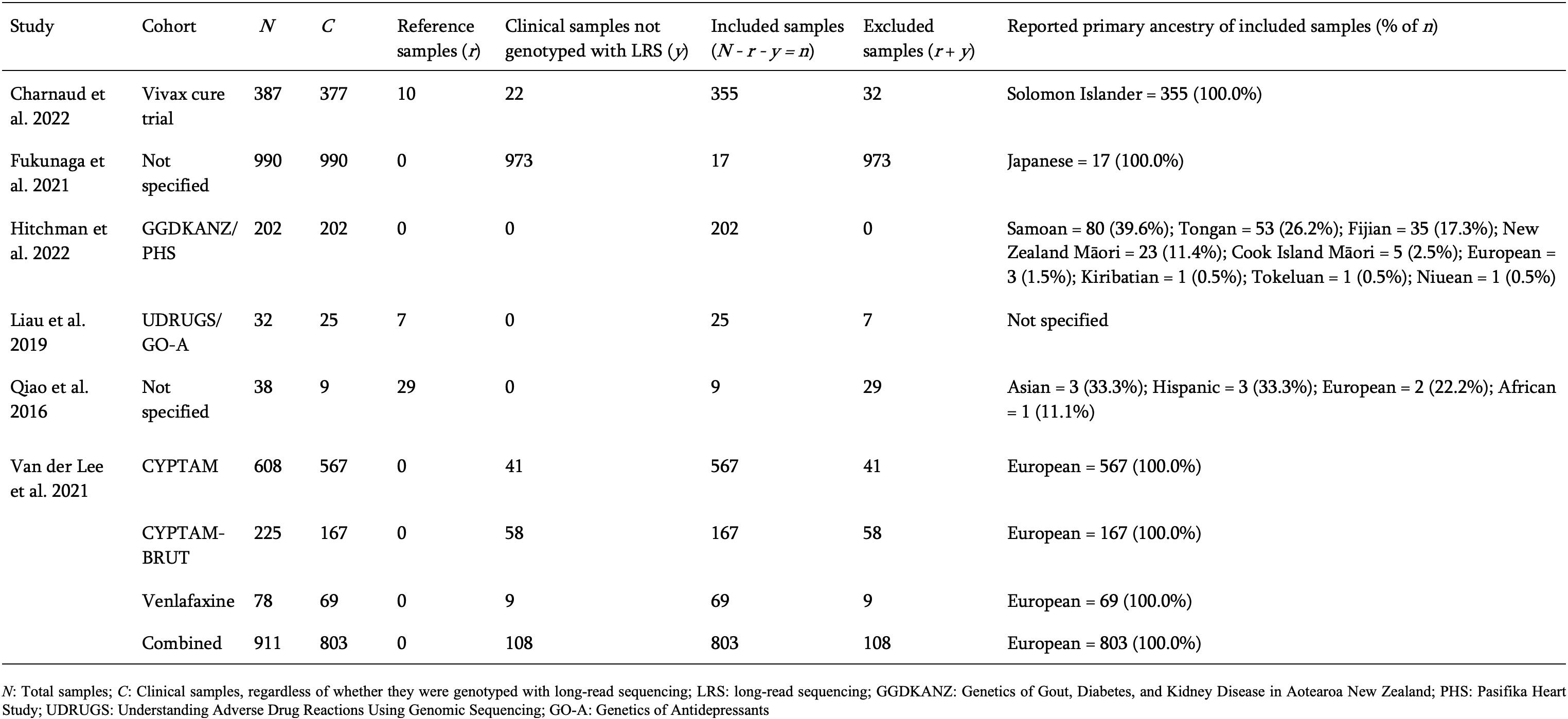

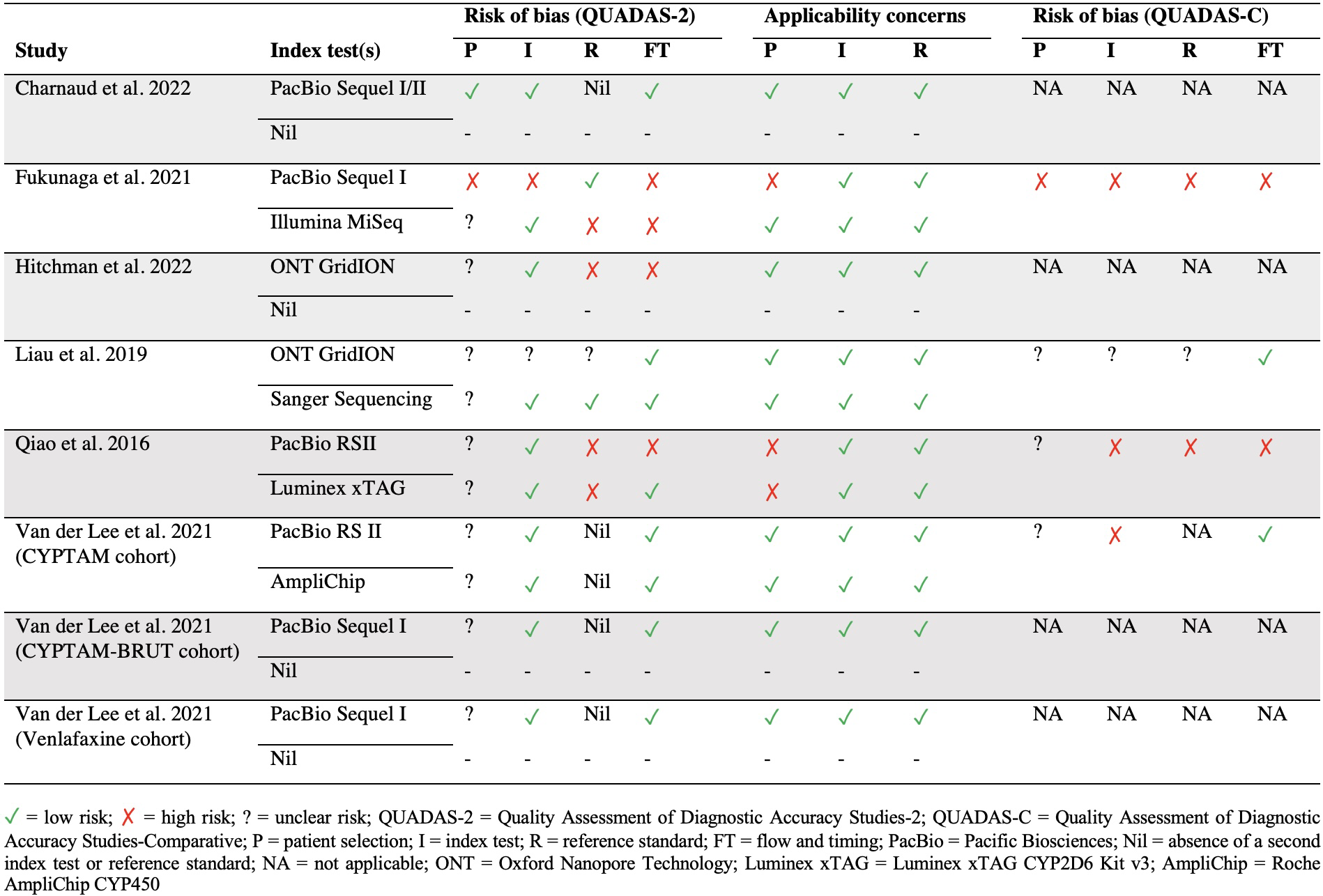

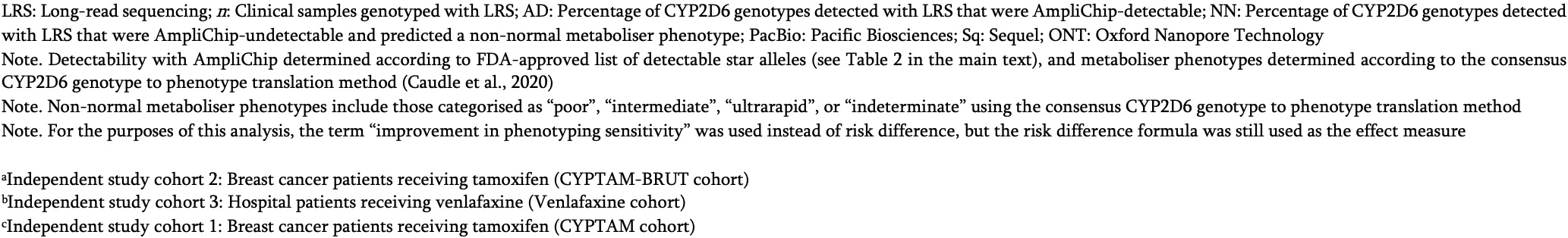

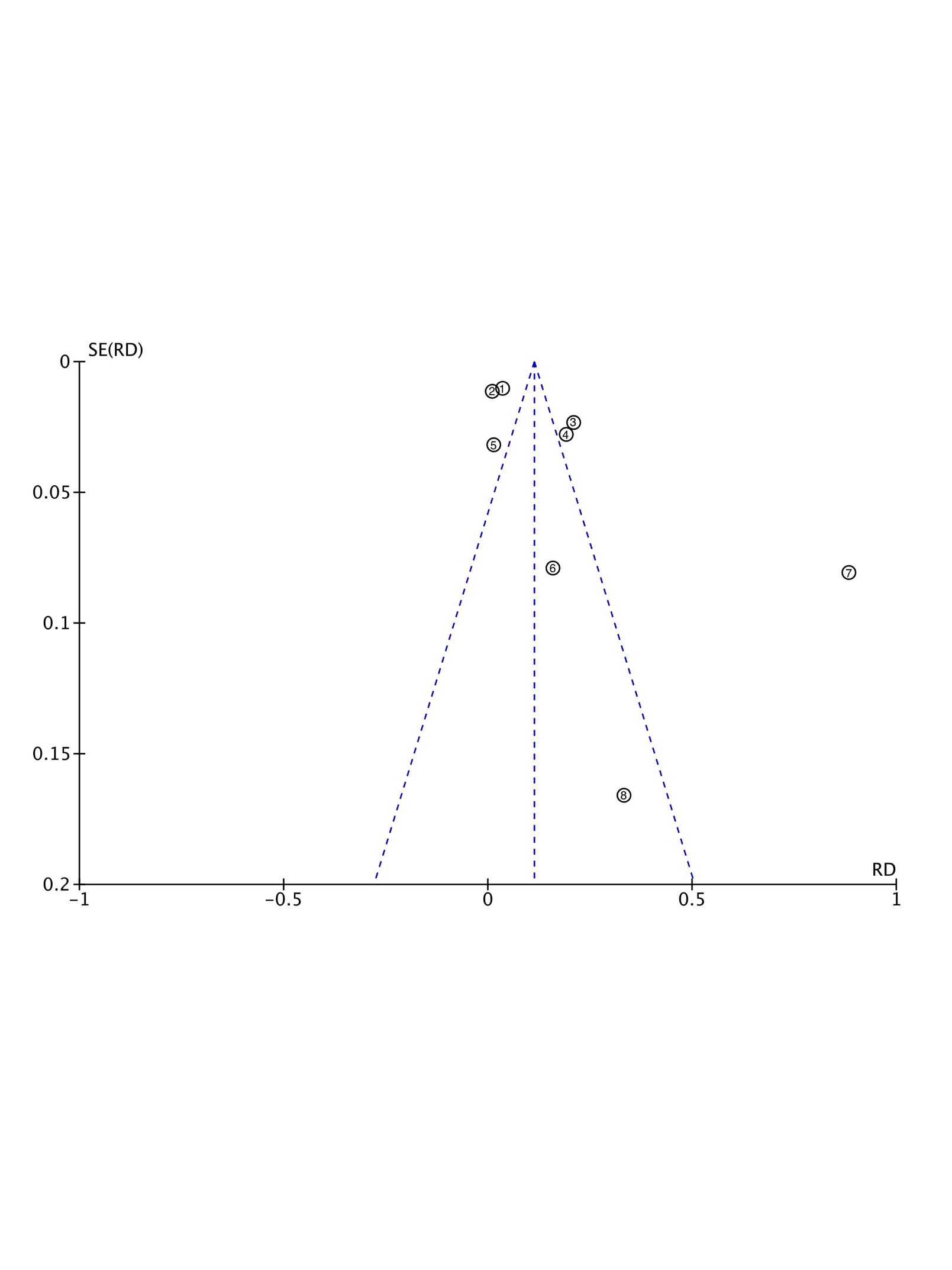


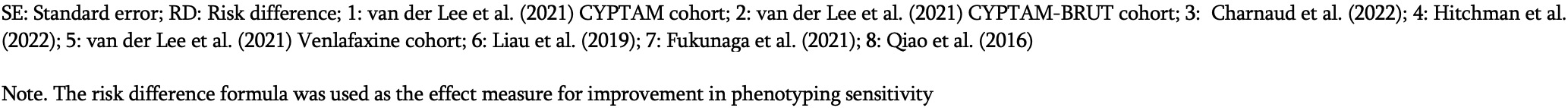

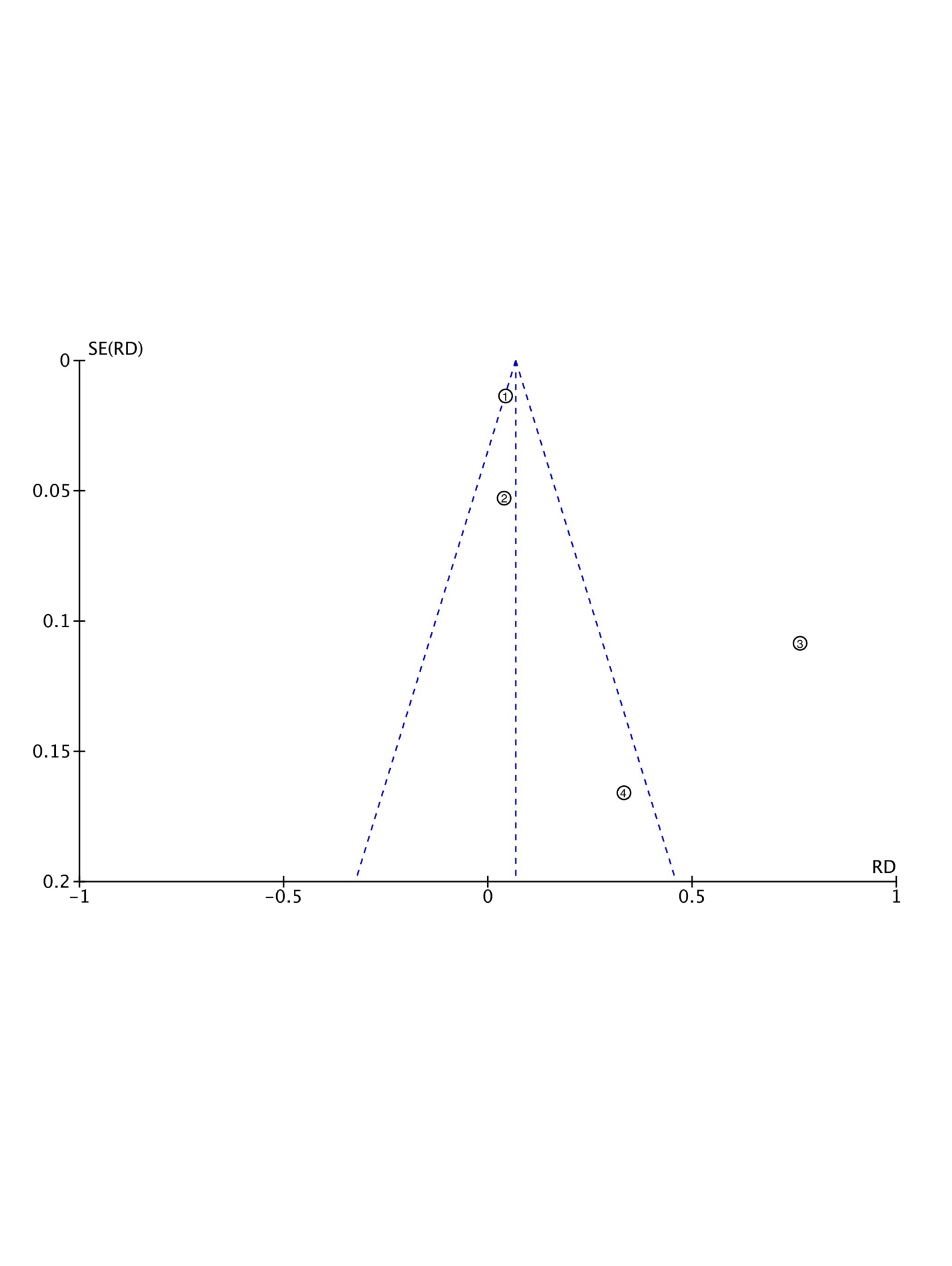


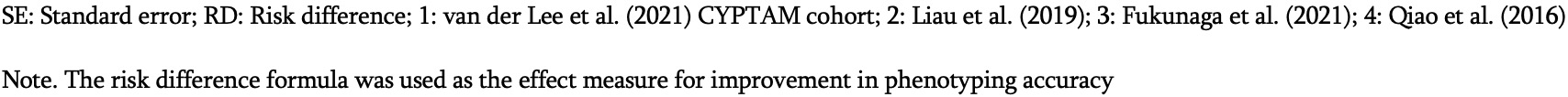


**References**

Caudle KE, Sangkuhl K, Whirl-Carrillo M, et al. (2020) Standardizing CYP2D6 Genotype to Phenotype Translation: Consensus Recommendations from the Clinical Pharmacogenetics Implementation Consortium and Dutch Pharmacogenetics Working Group. *Clin Transl Sci* 13(1): 116-124.

Charnaud S, Munro JE, Semenec L, et al. (2022) PacBio long-read amplicon sequencing enables scalable high-resolution population allele typing of the complex CYP2D6 locus. *Communications Biology* 5(1).

Fukunaga K, Hishinuma E, Hiratsuka M, et al. (2021) Determination of novel CYP2D6 haplotype using the targeted sequencing followed by the long-read sequencing and the functional characterization in the Japanese population. *Journal of Human Genetics* 66(2): 139-149.

Gaedigk A, Casey ST, Whirl-Carrillo M, et al. (2021) Pharmacogene Variation Consortium: A Global Resource and Repository for Pharmacogene Variation. *Clin Pharmacol Ther* 110(3): 542-545.

Hitchman LM, Faatoese A, Merriman TR, et al. (2022) Allelic diversity of the pharmacogene CYP2D6 in New Zealand Maori and Pacific peoples. *Front Genet* 13: 1016416.

Liau Y, Maggo S, Miller AL, et al. (2019) Nanopore sequencing of the pharmacogene CYP2D6 allows simultaneous haplotyping and detection of duplications. *Pharmacogenomics* 20(14): 1033-1047.

Page MJ, Mckenzie JE, Bossuyt PM, et al. (2021) The PRISMA 2020 statement: an updated guideline for reporting systematic reviews. *BMJ*. DOI: 10.1136/bmj.n71. n71.

Qiao W, Yang Y, Sebra R, et al. (2016) Long-Read Single Molecule Real-Time Full Gene Sequencing of Cytochrome P450-2D6. *Human Mutation* 37(3): 315-323.

van der Lee M, Allard WG, Vossen R, et al. (2021) Toward predicting CYP2D6-mediated variable drug response from CYP2D6 gene sequencing data. *Sci Transl Med* 13(603).

Whirl-Carrillo M, Huddart R, Gong L, et al. (2021) An Evidence-Based Framework for Evaluating Pharmacogenomics Knowledge for Personalized Medicine. *Clin Pharmacol Ther* 110(3): 563-572.
